# Studies Concerned with the Structure and Synthesis of the Anti‐viral Tropolone Glycoside Liriosmaside A

**DOI:** 10.1002/open.202500011

**Published:** 2025-04-08

**Authors:** Qi Chen, Yaping Zhan, Michael G. Gardiner, Zeinab G. Khalil, Amila A. Dewa, Thulasi Sritharan, Robert J. Capon, Ping Lan, Shen Tan, Martin G. Banwell

**Affiliations:** ^1^ Institute for Advanced and Applied Chemical Synthesis College of Pharmacy Jinan University Guangzhou 510632 China; ^2^ College of Pharmacy Jinan University Guangzhou 510632 China; ^3^ Research School of Chemistry Institute of Advanced Studies The Australian National University Canberra ACT 2601 Australia; ^4^ Institute for Molecular Bioscience University of Queensland St Lucia QLD 4072 Australia; ^5^ Anhui Jinhe Industrial Co. Ltd Chuzhou 239200 China

**Keywords:** tropone, ring-expansion, cross-coupling, glycosylation, anti-viral

## Abstract

A bromotropone corresponding to the agylcone of the glycosylated sesquiterpenoid natural product liriosmaside A has been prepared over ten steps and in a fully regio‐controlled manner through the *gem*‐dibromocyclopropane‐mediated ring‐expansion of a readily accessible decalenone. A Pd[0]‐mediated glucosylation reaction applied to this bromotropone afforded a product mixture from which an enantiomerically pure cross‐coupling product could be obtained and its structure confirmed through single‐crystal X‐ray analysis of a derivative. Various (unsuccessful) attempts are described to selectively acylate the last compound and thereby install the 3‐hydroxy‐3‐methylglutaric acid or HMGA‐containing side chain of the title natural product. A literature survey of other natural products embodying the HMGA motif suggest that liriosmaside A and its co‐metabolite liriosmaside B could be *S*‐configured at C3”. The evaluation of the glucosylated tropone in a series of anti‐bacterial, anti‐fungal and cytotoxicity assays reveals that it is inactive in all of these and so emphasizing the prospect that this and related troponoids, including the natural products liriosmaside A and B, can serve as useful models for new anti‐viral agents.

## Graphical abstract

The core of the anti‐viral troponoid natural product liriosmaside A lacking the 3‐hydroxy‐3‐methylglutaric acid (HMGA) side‐chain at C6′ has been prepared. The early steps involved the *gem*‐dibromocyclopropane‐mediated ring‐expansion of a readily‐obtained Robinson annulation product. These were followed by the Pd[0]‐catalysed glucosylation of the resulting racemic bromotropone and the structure of the chromatographically separated *R*‐isomer was established by single‐crystal X‐ray analysis.

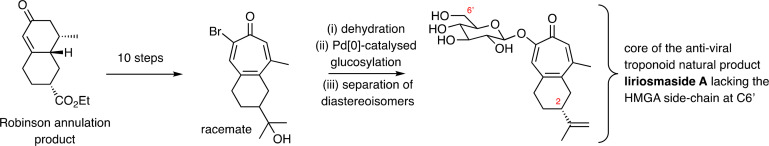

## Introduction

**Figure 1:**
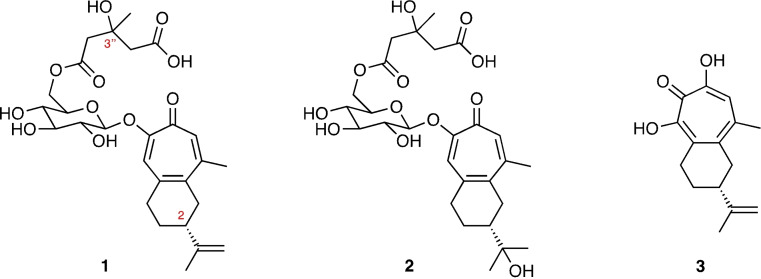
The structures of liriosmaside A (**1**), liriosmaside B (**2**) and manicol (**3**).

Recently there has been a resurgence of interest in troponoid compounds.^1^ This derives, in large part, from the recognition that more highly oxygenated and naturally‐occurring variants often display significant biological effects, most notably anti‐fungal, anti‐viral and anti‐malarial ones.[^1^, ^2^, ^3^] Liriosmasides A and B, **1** and **2** respectively (Figure 1), representing rare examples of glycosylated sesquiterpenoid tropolones, are cases in point.^[4]^ These were isolated from the tropical tree *Liriosma ovata* Meirs that is native to a number of Latin American countries and extracts from this and closely related species have been used as dietary supplements, for treating nervous disorders, as nerve stimulants and as aphrodisiacs. The structures of these natural products were proposed on the basis of extensive NMR spectroscopic and allied studies.^[4]^ *In vitro* testing of them revealed that the first was an inhibitor of HIV‐derived ribonuclease H (RNase H) activity (IC_50_=34 μM) although neither was active in a HIV cytopathicity assay up to 50 μM. In contrast, the structurally related natural product manicol (**3**)^[5]^ has been described as a potent and specific *in vitro* inhibitor of the same enzyme and, consequently, has been the subject of a number of derivatization studies as part of various efforts to identify even more active analogues.^[6]^

The development of total syntheses of targets such as troponoids **1** and **2** presents a number of significant challenges, notable amongst these being:

- the paucity of methodologies and strategies available for the selective assembly of specifically and highly substituted and/or carbannulated troponoids and especially ones incorporating centres of chirality within such frameworks;[^1^, ^7^, ^8^]
- The lack of processes available for the regio‐controlled glycosylation of troponoid compounds;
- The undefined stereochemistry at the stereogenic center (C3”) of the 3‐hydroxy‐3‐methylglutaric acid (HMGA) side‐chain and the need to confirm the assigned configuration at the isopropyl‐bearing carbon C2;
- The selective introduction of the double‐bond of the isopropenyl group without isomerization of this to the (presumably) thermodynamically more stable tetra‐substituted isomer;
- The general susceptibility of the troponoids toward ring‐contraction.

**Figure 2:**
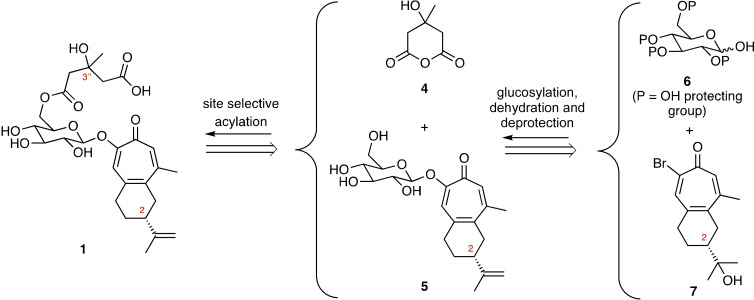
Retrosynthetic analysis employed in approaches to the synthesis of liriosmaside A (**1**).

In attempting to address these manifold issues, the implementation of the retrosynthetic analysis depicted in Figure 2 was pursued. This anticipated that final assembly of target **1** could be accomplished by reacting, in a regio‐selective manner, the known C_2_‐symmetric cyclic anhydride **4** with the glucosylated tropone **5**. Such a process was expected to deliver a mixture of target **1** and its C3”‐epimer and so offering the capacity to compare the spectral features of the two diastereoisomers and thereby complete the stereochemical details associated with the structure of the title natural product. Compound **5** was itself expected to be prepared through the β‐stereoselective coupling of a suitably protected glucose derivative **6** with the bromotropone **7** ^[9]^ then a dehydration reaction to establish the associated isopropenyl group. This would be followed by a deprotection step so as to reveal the four free hydroxyl residues associated with this sub‐target **5**.

## Results and Discussion

**Scheme 1:**
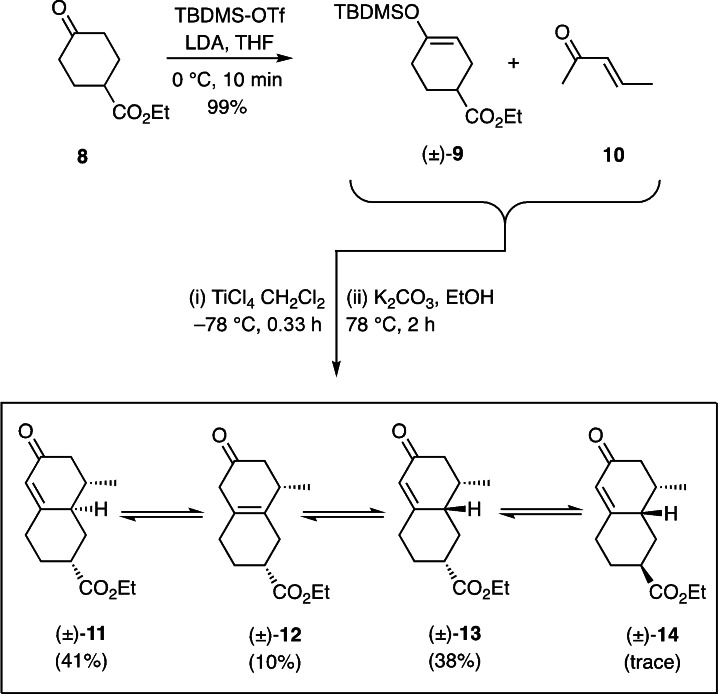
Syntheses of the isomeric decalenones (±)‐**11** to (±)‐**14** using a Mukaiyama Michael addition/Robinson annulation reaction.

The implementation of the synthetic plan shown in Figure 2 required access to the bromotropone **7** and amongst the modest number of methods available for the assembly of such highly substituted seven‐membered ring compounds, those involving the *gem*‐dibromocyclopropane‐mediated ring‐expansion of a suitably constituted decalin obtained using Robinson annulation techniques^[10]^ seemed most appropriate. The initial stages of our efforts in pursuit of such possibilities are shown in Scheme 1 and followed a previously established reaction sequence associated with our synthesis of the tropolone‐containing sesquiterpenoid olaximbriside A^[11]^ but details of which are shown again here for the sake of completeness.

Thus, the commercially available keto‐ester **8** was converted, under standard conditions, into the silyl enol ether (±)‐**9** (99 %) that was, in turn, engaged in a Michael addition reaction with methyl propenyl ketone (**10**) in the presence of TiCl_4_.^[12]^ The ensuing adduct was not isolated but immediately subjected to treatment with potassium carbonate in refluxing ethanol. As a result a chromatographically separable mixture of the previously unreported decalins (±)‐**11** to (±)‐**13** could be obtained (in 89 % combined yield) and each characterized independently, including through a single‐crystal X‐ray analysis of the last of these.^[11]^ Resubjection of compounds (±)‐**11** and (±)‐**12** to reaction with the same base for extended periods led to the formation of additional quantities of isomer (±)‐**13** although this was now becoming contaminated with another (inseparable) product tentatively identified as congener (±)‐**14**. Various efforts to render this Mukaiyama Michael addition/Robinson annulation reaction sequence enantioselective have been unsuccessful and so necessitating, as detailed below, resolution techniques be employed for accessing the desired enantiomeric form of sub‐target **5**.

**Scheme 2:**
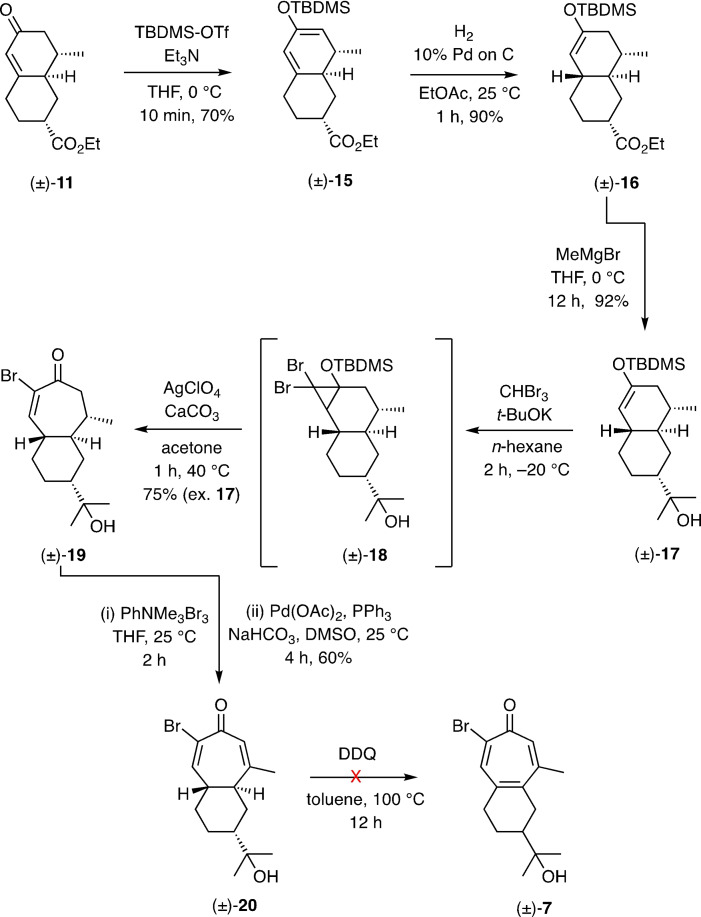
Attempted conversion of decalenone (±)‐**11** into 2‐bromotropone (±)‐**7** *via* a *gem*‐dibromocyclopropane‐mediated ring‐expansion reaction.

Since decalin (±)‐**11** proved to be the slightly more abundant isomer formed by the pathway described above, this was the one used in efforts to implement the foreshadowed ring‐expansion reaction. To such ends (Scheme 2), compound (±)‐**11** was converted into the corresponding silyl enol ether (±)‐**15** (70 %) under standard conditions and this, in turn, subjected to a 1,4‐hydrogenation^[13]^ using 10 % Pd on C as catalyst. As a result the new silyl enol ether (±)‐**16** (90 %) was obtained and the associated ester treated with an excess of methyl magnesium bromide to afford the 3°‐alcohol (±)‐**17** (92 %). Reaction of this last compound with dibromocarbene generated *in situ* by treating bromoform with *t*‐BuOK afforded what is presumed to be the ring‐fused *gem*‐dibromocyclopropane (±)‐**18** of undefined configuration. This adduct was immediately treated with silver perchlorate in the presence of calcium carbonate and so effecting electrocyclic ring‐opening with accompany collapse of the product π‐allyl‐cation through desilylation and thereby affording the bromobicyclo[5.4.0]undecenone (±)‐**19** [75 % ex. compound (±)‐**17**].^[14]^ α‐Bromination of compound (±)‐**19** with trimethylphenylammonium tribromide (PhNMe_3_Br_3_)^[15]^ in THF and treatment of the ensuing halogenated material with palladium(II) acetate, triphenylphosphine and sodium bicarbonate in DMSO at 25 °C^[16]^ afforded the dihydrotropone (±)‐**20** (60 %), the structure of which was confirmed by single‐crystal X‐ray analysis. However, all efforts to introduce the required, final degree of unsaturation into the seven‐membered ring of this compound, including through the use of DDQ, failed to deliver target (±)‐**7**. Attempts to effect an allylic bromination of compound (±)‐**20** (to be followed by dehydrobromination) using *N*‐bromosuccinimide (NBS), and azobisisobutyronitrile (AIBN) as radical initiator, only led to complex mixtures of products.

**Scheme 3:**
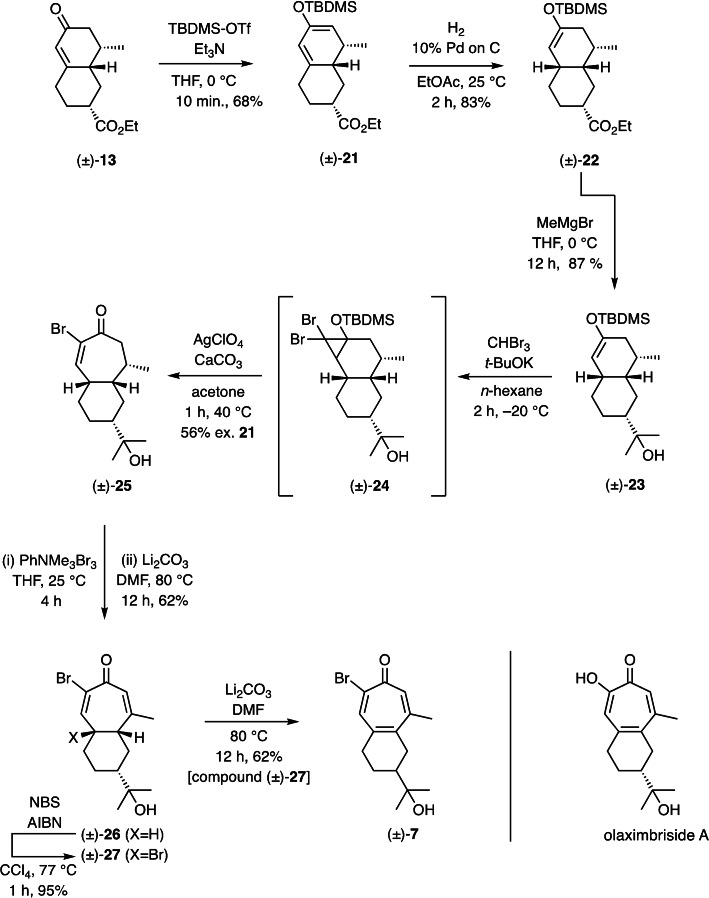
The conversion of decalin (±)‐**13** into 2‐bromotropone (±)‐**7**.

The difficulties just described in accessing target (±)‐**7** could be circumvented by repeating the same reaction sequence but now using, as shown in Scheme 3, decalin (±)‐**13** as the starting material. This sequence was established during our work on the synthesis olaximbriside A^[11]^ but is again presented here for the sake of completeness. All of the illustrated steps proceeded smoothly and culminated in the formation of the crystalline compound (±)‐**26**, the structure of which was confirmed, as previously reported,^[11]^ by single‐crystal X‐ray analysis. On subjecting this *cis*‐ring‐fused compound to allylic bromination with NBS and AIBN then a gum‐like dibromide was obtained in 95 % yield. Based on both spectroscopic and mechanistic considerations this is assigned as compound (±)‐**27** and when this was treated with lithium carbonate in DMF then the by now long sought‐after tropone (±)‐**7** ^[11]^ was obtained.

The divergent behaviors of the *trans*‐ and *cis*‐ring‐fused dihydrotropones (±)‐**20** and (±)‐**26**, respectively, under allylic bromination conditions is notable and the single‐crystal X‐ray analyses of each of these compounds provides some insights as to why this might be so. In particular, the allylic hydrogen to be abstracted within substrate (±)‐**26** is both sterically accessible and stereochemically well‐aligned with the adjacent π‐system while its counterpart in compound (±)‐**20**, and wherein the associated cyclohexane ring adopts a more chair‐like conformation, resides in a significantly more congested environment.

**Scheme 4:**
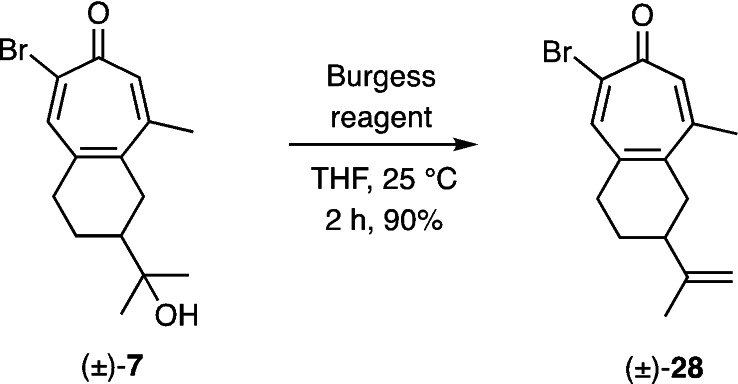
The regio‐controlled conversion of the 2‐bromotropone (±)‐**7** into its unsaturated counterpart (±)‐**28**.

The transformation of compound (±)‐**7** into the final target **1** requires, at some point, the dehydration of the 3°‐alcohol associated with the former compound. We chose to explore such possibilities using compound (±)‐**7** and after considerable experimentation it was established that this was best effected using the Burgess reagent^[17]^ at ambient temperatures and so affording the terminal alkene (±)‐**28** in 90 % yield (Scheme 4).

All of the spectral data recorded on troponoid (±)‐**28** [and indeed on precursor (±)‐**7**] were in complete accord with the assigned structures. Furthermore, the chemical shifts of the carbonyl carbons observed in the ^13^C NMR spectra of both these compounds (δ_C_ *ca*. 180) are closely aligned with those reported^[8c]^ for other analogously carbannulated 2‐bromotropones. The same is so for the C=O stretching bands (ν_max_ *ca*. 1610 cm^−1^) observed in the corresponding infra‐red spectra.

**Scheme 5:**
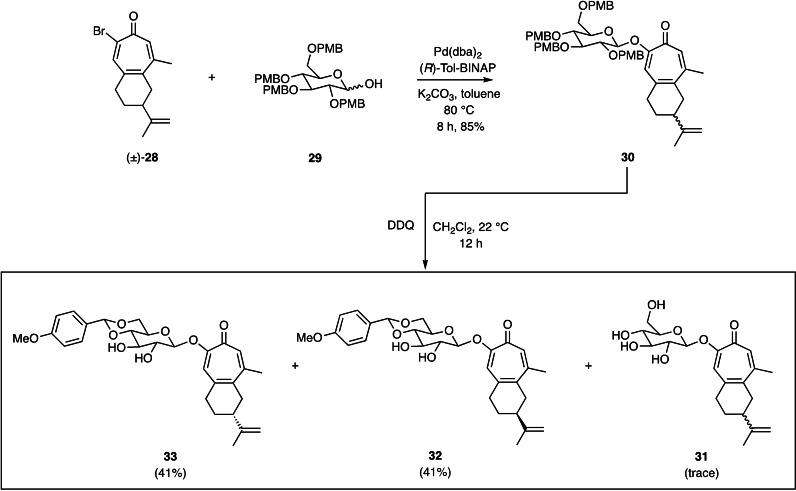
The Pd[0]‐catalyzed cross‐coupling of bromotropone **(**±)‐**28** with glucose derivative **29** leading, *via* product **30**, to the partially fully and partially deprotected glucosylated compounds **31**–**33**.

With compound (±)‐**7** to hand its coupling with a suitably protected glucose derivative could be pursued. The protocols we recently developed for such purposes^[18]^ and involving palladium‐catalyzed cross‐coupling chemistry proved very effective as shown in Scheme 5. In particular, reaction of compounds (±)‐**28** and **29** under the relevant conditions afforded the anticipated product **30** as a 1 : 1 mixture of diastereoisomers. On treatment of this mixture with DDQ in dichloromethane at ambient temperatures then the completely deprotected product **31** was obtained in trace amounts (and as 1 : 1 mixture of two diastereoisomers) with the partially deprotected, anisylidene acetal‐containing and chromatographically separable troponoids **32** and **33** being the predominant products (each obtained in 41 % yield). A single‐crystal X‐ray analysis of compound **33** served to establish that this, chromatographically less‐mobile isomer possessed the desired *R*‐configuration at the isopropenyl‐bearing carbon of the cyclohexane ring.

**Scheme 6:**
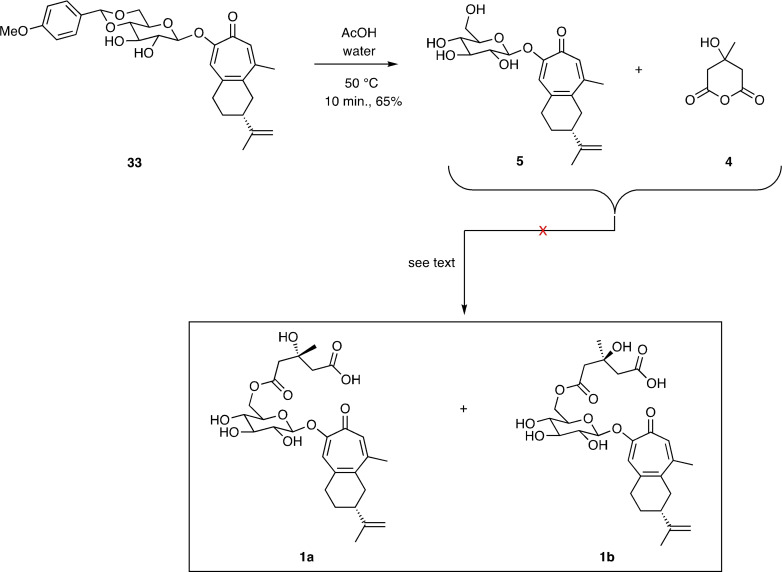
Attempted completion of total syntheses of the two possible epimeric forms, **1 a** and **1 b**, of liriosmaside A.

The synthesis of the glucosylated troponoid **5** proved straightforward and involved, as shown in Scheme 6, cleavage of the anisylidene‐acetal moiety associated with compound **33** using aqueous acetic acid at 50 °C for an abbreviated period of time. By such means compound **5** was obtained in 65 % yield. However, on treating this glucose derivative with the readily available C_2_‐symmetric cyclic anhydride **4** neither of the hoped‐for acylation products **1 a** or **1 b** was observed. Despite multiple attempts to implement this conversion only complex mixtures of uncharacterizable products were obtained, an outcome that may reflect the relatively poor electrophilicity of compound **4**.

**Scheme 7:**
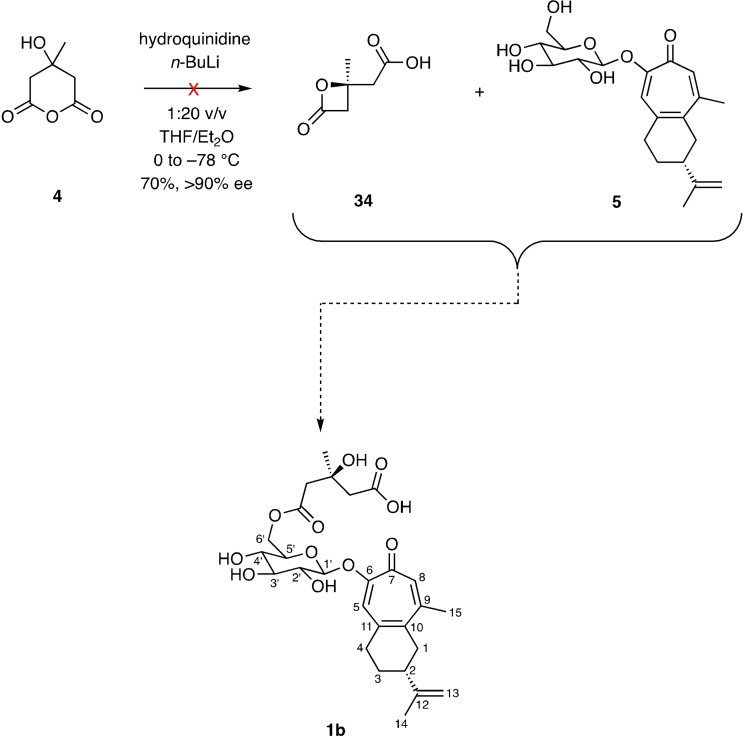
Attempted selective synthesis of compound **1 b** through formation and reaction of the β‐lactone **34** with glucoside **5**.

A literature survey of other natural products embodying the HMGA motif^[19]^ suggest that liriosmaside A (**1**) and congener B (**2**) might be *S*‐configured at C3”. Given this and the failure of the reaction sequence shown in Scheme 6, attempts were made to implement the one shown in Scheme 7. In particular, a protocol^[20]^ was investigated for desymmetrizing compound **4** (by deprotonation of this using the base derived from hydroquinidine and *n*‐BuLi) and so forming a potentially more effective electrophile, namely the *S*‐configured β‐lactone **34**. In the event, however, all efforts to prepare compound **34** in the indicated manner and by certain other means proved fruitless. As such the reaction of this β‐lactone with compound **5** could not be pursued. Consequently, we have been unable to complete a synthesis of the *S*‐configured C3′′‐compound **1 b**.

A comparison of the two sets of ^13^C{^1^H} NMR spectral data recorded on compounds **5** and liriosmaside A (**1**) is shown in Table 1 and reveals a good correlation (*viz*. relatively consistent Δδ_C_ values within the range of 1.0 to 1.80 ppm) except in those instances (see blue highlights) where the constituent (monosaccharide) carbons are associated (in the case of the natural product) with the HMGA side‐chain. Most conspicuously, then, the signal due to C6′ in liriosmaside A is significantly more deshielded (by 3.6 ppm) than its non‐acylated counterpart in compound **5**. Conversely, the signal due to C3′ in compound **5** is more deshielded that its counterpart in the natural product, an effect that may be due to intramolecular hydrogen bonding between the associated hydroxyl group and the HMGA side chain on the latter.

**Table 1:** Comparison of the ^13^C NMR chemical shifts reported^[4]^ for liriosmaside A (**1**) with those recorded on compound **5**

Assignment^a,b^	Liriosmaside A^c^ δ_C_	Compound 5^d^ δ_C_	Δδ_C_
C7	180.1	178.5	−1.6
C6	159.1	157.9	−1.2
C9	153.8	152.3	−1.5
C12	150.0	148.5	−1.5
C11	143.4	142.3	−1.1
C10	142.2	140.6	−1.6
C8	137.4	135.7	−1.7
C5	125.7	124.3	−1.4
C13	110.5	108.9	−1.6
C1′	101.8	100.8	−1.0
C3′	77.1	77.5	+0.4
C5′	75.9	75.9	0
C2′	74.5	73.1	−1.4
C4′	71.6	70.2	−1.4
C6′	64.9	61.3	−3.6
C2	42.1	40.6	−1.5
C4	38.0	36.2	−1.8
C1	37.2	35.7	−1.5
C3	28.0	26.6	−1.4
C15	27.4	25.8	−1.6
C14	21.0	19.5	−1.5

^a^ these assignments taken from ref. [4]; ^b^see structure **1 b** in Scheme 7 for carbon numbering; ^c^data {taken from ref. [4]} acquired in CD_3_OD at 150 MHz; ^d^data acquired in CD_3_OD at 150 MHz.

The specific rotations recorded for compound **5** and liriosmaside A were both laevorotatory and of similar magnitude {$[{\alpha ]}_{D}^{24\;}$ =−25.3 (*c*=0.36, methanol) and $[{\alpha ]}_{D}^{25}$ =−16.9 (*c*=0.21, methanol), respectively}.

## Outcomes of the Biological Testing of the Glucosylated Tropone 5

If compound **5** and its congeners, including natural products **1** and **2**, are to be developed as leads for identifying new anti‐viral agents then they are most likely to be useful for such purposes if they lack cytotoxic and anti‐bacterial effects (the latter meaning they would be less likely to display adverse gastrointestinal impacts under oral administration regimes). In anti‐bacterial assays, the compound was tested against Gram‐positive *Staphylococcus aureus* ATCC25923, Gram‐negative *Escherichia coli* ATCC11775 as well as clinical isolates of extended‐spectrum β‐lactamase (ESBL)‐resistant *Escherichia coli*, methicillin‐resistant *Staphylococcus aureus* (MRSA, AUS‐RBWH‐MRSA‐02) and vancomycin‐resistant *Enterococci*. In an anti‐fungal assay compound **5** was tested against *Candida albicans* ATCC10231 while cytotoxicity assessments were conducted using the MTT assay on SW620 human colorectal carcinoma cells, with doxorubicin as the positive control. In all of these tests compound **5** did not exhibit any significant anti‐bacterial, anti‐fungal or cytotoxic activities.

## Conclusions

A fully regio‐controlled synthesis of the carbocyclic and glycosylated framework, **5**, of the sesquiterpenoid and tropone‐containing natural product liriosmaside A (**1**) has been achieved. However, all efforts to attach the 3‐hydroxy‐3‐methylglutaric acid or HMGA residue to compound **5** and thereby establish the configuration at C3“ in natural product **1** have been unsuccessful. Nevertheless, based on biosynthetic considerations, the *S*‐configuration could be proposed for this stereogenic center in liriosmaside A (*viz*. its structure is represented by **1 b**). The biological evaluation of compound **5** in a range of anti‐bacterial, anti‐fungal and cytotoxicity assays reveal that this glucosylated tropone is inactive. This thus augers well for the development of such compounds as anti‐viral agents.

## Supporting Information

Experimental protocols for the the synthesis of compounds (±)‐**15**, (±)‐**16**, (±)‐**17**, (±)‐**19**, (±)‐**20**, (±)‐**28**, **30**, **32**, **33** and **5**, plots derived from the single crystal X‐ray analyses of compounds (±)‐**20** and **33** together with copies of the ^1^H and ^13^C{^1^H} NMR spectra of compounds (±)‐**15**, (±)‐**16**, (±)‐**17**, (±)‐**19**, (±)‐**20**, (±)‐**28**, (±)**‐7, 30**, **32**, **33** and **5** (PDF). X‐ray crystallographic data for compounds (±)‐**20** and **33** (CIFs).

Deposition numbers 2091407 for [(±)‐**20**] and 2388550 (for **33**) contain the supplementary crystallographic data for this paper. These data are provided free of charge by the joint Cambridge Crystallographic Data Centre and Fachinformationszentrum Karlsruhe (http://www.ccdc.cam.ac.uk/structures).

## Conflict of Interests

The authors declare no competing financial interest.

## Supporting information

As a service to our authors and readers, this journal provides supporting information supplied by the authors. Such materials are peer reviewed and may be re‐organized for online delivery, but are not copy‐edited or typeset. Technical support issues arising from supporting information (other than missing files) should be addressed to the authors.

Supporting Information

## Data Availability

The data that support the findings of this study are available from the corresponding author upon reasonable request.
