# Supplementary material for: Studies Concerned with the Structure and Synthesis of the Anti‐viral Tropolone Glycoside Liriosmaside A
Source: ChemistryOpen. 2025 Apr 8;14(8):e202500011. doi: 10.1002/open.202500011 (PMC12368893; doi:10.1002/open.202500011)

ChemistryOpen

Supporting Information

Studies Concerned with the Structure and Synthesis of the Anti-viral Tropolone Glycoside Liriosmaside A

Qi Chen, Yaping Zhan, Michael G. Gardiner, Zeinab G. Khalil, Amila A. Dewa, Thulasi Sritharan, Robert J. Capon, Ping Lan, Shen Tan,* and Martin G. Banwell*

SUPPORTING INFORMATION FOR:

Studies Concerned with the Structure and Synthesis of the Anti-viral Tropolone Glycoside Liriosmaside A

Qi Chen,[a] Yaping Zhan,[b] Michael G. Gardiner,[c] Zeinab G. Khalil,[d]

Amila Agampodi Dewa,[d] Thulasi Sritharan,[d] Robert J. Capon,[d] Ping Lan[a,e]

Shen Tan[a,]* and Martin G. Banwell[a,]*

[a]Institute for Advanced and Applied Chemical Synthesis, The State Key Laboratory of Bioactive Molecules and Druggability Assessment and The Guangdong Province Key Laboratory of Pharmacodynamic Constituents of TCM & New Drugs Research, College of Pharmacy, Jinan University, Guangzhou, 510632, China

and

[b]College of Pharmacy, Jinan University, Guangzhou, 510632, China

and

[c]Research School of Chemistry, Institute of Advanced Studies, The Australian National University, Canberra, ACT 2601, Australia

and

[d]Institute for Molecular Bioscience, University of Queensland

St Lucia, QLD 4072, Australia

and

[e]Anhui Jinhe Industrial Co. Ltd, Chuzhou, 239200, China

Table of Contents

(i) Experimental Proceedures;

(ii) **Figures S1 and S2**: Plots derived from the single-crystal X-ray analyses of compounds **20** and **33**;

(iii) ^{1}H and ^{13}C{^{1}H} NMR spectra of compounds (±)-**15**, (±)-**16**, (±)-**17**, (±)-**19**, (±)-**20**, (±)-**28**, **30**, **32**, **33** and **5**.

Experimental

General Experimental Protocols

Unless otherwise specified, proton (^{1}H) and carbon (^{13}C) NMR spectra were recorded at room temperature in base-filtered $CDCl_3$ on a spectrometer operating at 300 MHz for proton and 75 MHz for carbon nuclei. For ^{1}H NMR spectra, signals arising from the residual protioforms of the solvent were used as the internal standards. ^{1}H NMR data are recorded as follows: chemical shift (δ) [multiplicity, coupling constant(s) *J* (Hz), relative integral] where multiplicity is defined as: s = singlet; d = doublet; t = triplet; q = quartet; m = multiplet or combinations of the above. The signal due to residual $CHCl_3$ appearing at δ_H 7.26 and the central resonance of the $CDCl_3$ "triplet" appearing at δ_C 77.0 were used to reference ^{1}H and ^{13}C NMR spectra, respectively. Infrared spectra were recorded, as thin films or solids, on a Nicolet iS50 FT-IR spectrometer fitted with a Smart iTX sampling module. High-resolution ESI mass spectra were recorded on a time-of-flight instrument. Melting points were measured on an automated melting point system and are uncorrected. Analytical thin layer chromatography (TLC) was performed on aluminium-backed 0.2 mm thick silica gel 60 F254 plates. Eluted plates were visualized using a 254 nm UV lamp and/or by treatment with a suitable dip followed by heating. These dips included phosphomolybdic acid: ceric sulfate : sulfuric acid (conc.) : water (37.5 g : 7.5 g : 37.5 g : 720 mL) or potassium permanganate : potassium carbonate : 5% sodium hydroxide aqueous solution : water (3 g : 20 g: 5 mL : 300 mL). Flash chromatographic separations were carried out following protocols defined by Still *et al.*[1] with silica gel 60 (40–63 µm) as the stationary phase and using the AR- or HPLC-grade solvents indicated. Petroleum ether refers to the fraction boiling between 40 and 60 °C. Starting materials, reagents and drying agents as well as other inorganic salts were generally available from commercial sources and used as supplied. Tetrahydrofuran (THF), diethyl ether, methanol and dichloromethane were dried using a solvent purification system that is based upon a technology originally described by Grubbs *et al.*[2] Where necessary, reactions were performed under a nitrogen atmosphere. HPLC was performed on a Shimadzu LC20A instrument fitted with a UV detection system and a Cosmosil 5 µM C18-MS-II 120 Å 250 × 20 mm column that was eluted with water/methanol.

Specific Chemical Transformations

Compound (±)-15

A magnetically stirred solution of decalin (±)-**11**[3] (5.00 g, 21.2 mmol) in dry THF (100 mL) maintained at 0 °C under a nitrogen atmosphere was treated with triethylamine (8.8 mL, 63.6 mmol) and then, dropwise, with TBSOTF (7.30 mL, 31.8 mmol). The resulting mixture was stirred for 10 min at 0 °C then quenched with $NaHCO_3$ (100 mL of a saturated aqueous solution) before being extracted with petroleum ether (3 × 100 mL of the 40-60 fraction). The combined organic layers were washed with water (2 × 100 mL) and brine (2 × 100 mL) then dried (Na_2SO_4), filtered and concentrated under reduced pressure. The residue so-obtained was subjected to flash chromatography (silica, 10:1 v/v petroleum ether/ethyl acetate elution) to afford, after concentration of the appropriate fractions (R_f = 0.9 in 30:1 v/v petroleum ether/ethyl acetate), compound (±)-**15** (5.10 g, 70%) as a clear, light-green oil. ^{1}H NMR (300 MHz, $CDCl_3$) δ 5.33 (d, *J* = 1.4 Hz, 1H), 5.25 (m, 1H), 4.25-4.04 (complex m, 2H), 2.86 (m, 1H), 2.58 (m, 1H), 2.42 (m, 1H), 2.26 (m, 1H), 2.08 (m, 1H), 1.96 (m, 1H), 1.78 (m, 1H), 1.49 (m, 1H), 1.37 (m, 1H), 1.25 (m, 3H), 1.01 (d, *J* = 6.5, 3H), 0.92 (s, 9H), 0.16 (s, 3H), 0.15 (s, 3H); ^{13}C{^{1}H} NMR (75 MHz, $CDCl_3$) δ 175.1, 151.9, 136.5, 116.8, 109.0, 60.2, 39.7, 37.6, 37.1, 34.8, 28.7, 26.4, 25.6, 19.0, 18.0, 14.3, –4.2, –4.4; IR ν_{max} 2956, 2930, 1725, 1665, 1461, 1372, 1252, 1189, 1065,1028 cm^{-1}; HRMS (ESI, +ve) *m/z* $(M+H)^+$ calcd for $C_{20}H_{35}O_3Si$ 351.2350, found 351.2340.

Compound (±)-16

A magnetically stirred solution of diene (±)-**15** (3.00 g, 8.6 mmol) in ethyl acetate (150 mL) maintained at 25 °C under a nitrogen atmosphere was treated with 10% palladium on carbon (150 mg). The nitrogen atmosphere was replaced, via balloon, with hydrogen and the reaction mixture stirred for *ca*. 1 h (careful monitoring by TLC required to avoid over-reduction) then filtered through a pad of Celite®. The solids thus retained were washed with ethyl acetate and the combined filtrates concentrated under reduced to afforded compound (±)-**16** (2.70 g, 90%) as a clear, colorless oil. This material was used without further purification in the next step of the reaction sequence. ^{1}H NMR (300 MHz, $CDCl_3$) δ 4.60 (broad s, 1H), 4.18-4.11 (complex m, 2H), 2.76 (m, 1H), 2.36 (m, 1H), 2.19 (m, 1H), 2.11-1.94 (complex m, 2H), 1.92-1.69 (complex m, 3H), 1.64-1.41 (complex m, 4H), 1.31-1.22 (complex m, 3H), 1.09 (d, *J* = 6.5, 3H), 0.91 (s, 9H), 0.12 (s, 6H); ^{13}C{^{1}H} NMR (75 MHz, $CDCl_3$) δ 175.3,

149.8, 109.4, 60.2, 42.9, 41.2, 40.0, 39.8, 34.0, 30.8, 30.4, 27.4, 25.8, 18.7, 18.1, 14.4, –4.2, –4.4; IR ν_{max} 2953, 2924, 2855, 1730, 1465, 1442, 1367, 1257, 1164, 1143 cm^{-1}; HRMS (ESI, +ve) *m/z* $(M+H)^+$ calcd for $C_{20}H_{37}O_3Si$ 353.2506, found 353.2503.

Compound (±)-17

A magnetically stirred solution of ester **(±)-16** (2.00 g, 5.7 mmol) in dry THF (50 mL) maintained at 0 °C under a nitrogen atmosphere was treated, dropwise, with methylmagnesium bromide (5.70 mL of a 3.0 M in diethyl ether, 17.1 mmol). After the addition was complete the reaction mixture was allowed to warmed to 25 °C then stirred for a further 12 h before being quenched with ammonium chloride (50 mL of a saturated aqueous solution) and extracted with ethyl acetate (3 × 100 mL). The combined organic layers were washed with water (2 × 100 mL) and brine (2 × 100 mL) then dried (Na_2SO_4), filtered and concentrated under reduced pressure. The residue mixture thus obtained was subjected to flash chromatography (silica, 10:1 v/v petroleum ether/ethyl acetate elution) to afford, after concentration of the appropriate fractions (R_f = 0.6 in 10:1 v/v petroleum ether/ethyl acetate), compound **(±)-17** (1.70 g, 90%) as a clear, colorless oil. ^1H NMR (300 MHz, $CDCl_3$) δ 4.66 (s, 1H), 2.17 (m, 1H), 1.98 (m, 1H), 1.92-1.74 (complex m, 3H), 1.73-1.47 (complex m, 5H), 1.44-1.32 (complex m, 2H), 1.28 (s, 6H), 1.18 (s, 1H), 0.99 (d, *J* = 6.7 Hz, 3H), 0.93 (s, 9H), 0.14 (s, 6H); $^{13}C\{^1H\}$ NMR (75 MHz, $CDCl_3$) δ 149.3, 109.6, 73.9, 42.9, 40.8, 39.5, 37.5, 35.2, 30.5, 29.3, 29.0, 28.1, 25.8, 23.4, 18.7, 18.1, –4.2, –4.4; IR ν_{max} 3390, 2927, 2856, 1461, 1366, 1251, 1196, 1146, 1104, 1074 cm^{-1}; HRMS (ESI, +ve) *m/z* $(M+H)^+$ calcd for $C_{20}H_{39}O_2Si$ 339.2714, found 339.2703.

Compound (±)-19

A solution of bromoform (480 μL, 5.56 mmol) in hexane (3 mL) was added, dropwise, to a magnetically stirred mixture of 3°-alcohol **(±)-17** (235 mg, 0.7 mmol) and *t*-BuOK (778 mg, 7.0 mmol) in hexane (10 mL) maintained at –20 °C. After addition was complete, stirring was continued at –20 °C for 2 h then the reaction mixture was filtered through a pad of TLC-grade silica. The solids thus retained were washed a mixture of petroleum ether/ethyl acetate (70 mL of 5:2 v/v mixture) and the combined filtrates concentrated under reduced pressure. The red oil thus obtained was dissolved in acetone (10 mL) then treated with Ca_2CO_3 (659 mg, 6.6 mmol) and $AgClO_4$ (351 mg, 1.7 mmol) and the resulting mixture stirred at 40 °C for 1 h before being cooled to room temperature then treated with triethylamine (500 μL) followed by petroleum ether (30 mL). The

resulting mixture was filtered through a pad of TLC-grade silica and the solids thus retained were washed with petroleum ether/ethyl acetate (100 mL of a 1:1 v/v mixture). The combined filtrates were concentrated under reduced pressure and the residue so produced subjected to flash chromatography (silica, 20:1 → 3:1 v/v petroleum ether/ethyl acetate gradient elution) to afford, after concentration of the appropriate fractions (R_f = 0.3 in 3:1 v/v petroleum ether/ethyl acetate), compound (±)-**19** (167 mg, 76%) as a clear, light-green oil. ^{1}H NMR (300 MHz, $CDCl_3$) δ 7.26 (d, *J* = 3.4 Hz, 1H), 2.98 (dd, *J* = 12.4 and 6.6 Hz, 1H), 2.65 (dd, *J* = 12.4 and 2.7 Hz, 1H), 2.44 (m, 1H), 2.28 (broad s, 1H), 1.88-1.78 (complex m, 3H), 1.71-1.64 (complex m, 3H), 1.50-1.30 (complex m, 3H), 1.20 (s, 3H), 1.17 (s, 3H), 1.03 (d, *J* = 6.7 Hz, 3H); ^{13}C{^{1}H} NMR (75 MHz, $CDCl_3$) δ 193.8, 157.8, 126.9, 73.3, 47.9, 46.5, 41.0, 40.1, 34.2, 28.7, 28.3, 27.4, 26.5, 23.1, 19.8; IR ν_{max} 3462, 2961, 2871, 1674, 1457, 1366, 1228, 1182, 1151, 1044 cm^{-1}; HRMS (ESI, +ve) *m/z* $(M+H)^+$ calcd for $C_{15}H_{24}{}^{79}BrO_2$ 315.0954, found 315.0957.

Compound (±)-20

A magnetically stirred solution of enone (±)-**19** (67 mg, 0.21 mmol) in THF (5 mL) maintained at 25 °C under a nitrogen atmosphere was treated, in one portion, with phenyltrimethylammonium tribromide (160 mg, 0.42 mmol). After 2 h the reaction mixture was quenched with $Na_2S_2O_3$ (10 mL of a 0.1 M aqueous solution) and extracted with ethyl acetate (3 × 10 mL). The combined organic phases were washed with water (2 × 10 mL) and brine (2 × 10 mL) before being dried (Na_2SO_4), filtered and concentrated under reduced pressure. The residue thus obtained was dissolved in DMSO (10 mL) and the resulting solution treated, while being maintained under a nitrogen atmosphere, with PPh_3 (30 mg, 0.12 mmol) and $NaHCO_3$ (35 mg 0.4 mmol). After purging the resulting mixture with nitrogen for 10 min $Pd(OAc)_2$ (14 mg, 0.06 mmol) was added. After 4 h, the reaction mixture was quenched with water (20 mL) and extracted with ethyl acetate (3 × 20 mL). The combined organic layers were washed with water (2 × 20 mL) and brine (2 × 20 mL) then dried (Na_2SO_4), filtered and concentrated under reduced pressure. The residue thus obtained was subjected to flash chromatography (silica, 2:1 v/v petroleum ether/ ethyl acetate elution) to afford, after concentration of the appropriate fractions (R_f = 0.4 in 2:1 v/v petroleum ether/ethyl acetate), compound (±)-**20** (40 mg 60%) as a colorless, crystalline solid, m.p. = 99-100 °C. ^{1}H NMR (300 MHz, $CDCl_3$) δ 7.12 (d, *J* = 3.8 Hz, 1H), 6.21 (m, 1H), 2.86 (m, 1H), 2.59 (m, 1H), 2.08 (t, *J* = 1.2 Hz, 3H), 1.80-1.49 (complex m, 8H), 1.25

(s, 3H), 1.21 (s, 3H); $^{13}C\{^1H\}$ NMR (75 MHz, $CDCl_3$) δ 184.6, 160.8, 152.4, 128.6, 126.2, 73.4, 41.2, 40.4, 40.0, 28.7, 28.1, 26.6, 24.8, 24.1, 22.6; IR ν_{max} 3239, 2960, 2924, 1620, 1599, 1461, 1412, 1354, 1325, 1261, 1237, 1218, 1181, 1145 cm^{-1}; HRMS (ESI, +ve) *m/z* $(M+H)^+$ calcd for $C_{15}H_{22}{}^{79}BrO_2$ 313.0798, found 313.0807.

Compound (±)-28

A magnetically stirred solution of tropone (±)-**7**[3] (50 mg, 0.16 mmol) in dry THF (5 mL) and maintained under a nitrogen atmosphere was treated with (methoxycarbonylsulfamoyl)-triethylammonium hydroxide (Burgess reagent) (76 mg, 0.32 mmol). The ensuing mixture was stirred at 25 °C for 2 h before being quenched with water (10 mL) then extracted with ethyl acetate (3 × 10 mL). The combined organic layers were washed with water (2 × 10 mL) and brine (2 × 10 mL) before being dried (Na_2SO_4), filtered and concentrated under reduced pressure. The residue thus obtained was subjected to flash chromatography (silica, 10:1 v/v petroleum ether/ ethyl acetate elution) to afford, after concentration of the appropriate fractions (R_f = 0.3 in 5:1 v/v petroleum ether/ethyl acetate), compound (±)-**28** (42 mg, 90%) as a yellow powder, m.p. = 121-122 °C. ^1H NMR (300 MHz, $CDCl_3$) δ 7.87 (s, 1H), 7.14 (s, 1H), 4.85 (s, 1H), 4.77 (s, 1H), 3.05-2.77 (complex m, 2H), 2.71 (m, 1H), 2.46 (m, 1H), 2.31 (s, 3H), 2.25 (m, 1H), 1.98 (m, 1H), 1.82 (s, 3H), 1.60 (m, 1H); $^{13}C\{^1H\}$ NMR [75 MHz, $(CD_3)_2SO$] δ 177.8, 149.1, 148.2, 143.6, 143.1, 138.8, 135.6, 134.3, 109.8, 39.7, 35.2, 34.5, 26.0, 25.6, 20.5; IR ν_{max} 3455, 2959, 2932, 1611, 1564, 1494, 1446, 1413, 1375, 1186 cm^{-1}; HRMS (ESI, +ve) *m/z* $(M+H)^+$ calcd for $C_{15}H_{18}{}^{79}BrO$ 293.0536, found 293.0528.

Compound 30

An oven-dried Schlenk tube was charged with compound (±)-**28** (29.5 mg, 0.1 mmol), $Pd(dba)_2$ (5.7 mg, 0.01 mmol), BINAP (13 mg, 0.02 mmol), K_2CO_3 (21 mg, 0.15 mmol) and compound **29**[4] (72 mg, 0.11 mmol). Air was removed from the Schlenk tube under reduced pressure and replaced by nitrogen three times before deoxygenated toluene (600 μL) was added via syringe. The ensuing mixture was stirred at 100 °C (oil-bath) for 8 h then cooled, diluted with water (10 mL) and the separated aqueous phase extracted with ethyl acetate (3 × 20 mL). The combined organic phases were washed with water (2 × 20 mL) and brine (2 × 20 mL) before being dried (Na_2SO_4), filtered and concentrated under reduced pressure. The

ensuing residue was subjected to flash chromatography (silica, 1:2 v/v petrol ether/ ethyl acetate elution) to afford, after concentration of the appropriate fractions (R_f = 0.2 in 1:1 v/v petrol ether/ethyl acetate), compound **30** (31 mg, 85%) as a clear, yellow oil and as a *ca*. 1:1 mixture of diastereoisomers. ^{1}H NMR (300 MHz, $CDCl_3$) δ 7.38 (m, 2H), 7.34-7.08 (complex m, 6H), 6.96 (s, 1H), 6.93-6.77 (complex m, 9H), 5.27 (d, *J* = 7.6 Hz, 0.5H), 5.24 (d, *J* = 7.6 Hz, 0.5H), 5.03-4.68 (complex m, 7H), 4.55-4.35 (complex m 3H), 3.83 (s, 3H), 3.82 (s, 3H), 3.80 (m, 6H), 3.76-3.66 (complex m, 2.5H), 3.62-3.46 (complex m, 3.5H), 2.80-2.50 (complex m, 3H), 2.43 (m, 1H), 2.32 (s, 3H), 2.19 (m, 1H), 1.81 (s, 3H), 1.79-1.69 (complex m, 2H); ^{13}C NMR (75 MHz, $CDCl_3$) δ 178.5, 178.4, 159.3, 159.2(2), 159.1(8), 158.3, 158.1, 148.7, 148.6(2), 148.5(6), 148.4(8), 139.4, 139.2, 138.4(0), 138.3(5), 137.3(1), 137.2, 130.8 130.7, 130.4(4), 130.4(1), 130.2, 130.1, 129.6(6), 129.5(8), 129.5(2), 123.4, 123.2, 113.8, 113.7(3), 113.6(8), 113.6(5), 109.8, 101.5, 101.4, 84.2, 81.1, 75.4, 74.7, 74.4, 73.2, 68.9, 55.3(1), 55.2(5), 55.2(1), 40.8, 40.6, 36.3, 36.1, 35.8(4), 35.7(7), 26.8, 26.7, 26.6, 20.7(4), 20.6(9) (25 signals obscured or overlapping); IR ν_{max} 2926, 2835, 1611, 1512, 1463, 1244, 1066, 1032, 819 cm^{-1}; HRMS (ESI, +ve) *m/z* $(M+H)^+$ calcd for $C_{53}H_{61}O_{11}$ 873.4208, found 873.4196.

Compounds 32 and 33

A magnetically stirred solution of compound **30** (20.0 mg, 0.023 mmol) in dry dichloromethane (2 mL) maintained at ambient temperatures was treated with DDQ (21 mg, 0.092 mmol). After 12 h the reaction mixture was treated with water (10 mL) and the separated aqueous phase extracted with dichloromethane (3 × 10 mL). The combined organic phases were then washed with water (2 × 10 mL) and brine (2 × 10 mL) before being dried (Na_2SO_4), filtered and concentrated under reduced pressure. The residue thus obtained was subjected to reverse-phase preparative HPLC (73:27 v/v MeOH/ water elution, flow rate = 8 mL/min) to afford two fractions, A and B.

Concentration of fraction A (t_R = 53 min) gave compound **32** (6.70 mg, 41%) as white crystals, m. p. = 108-109 °C, $[\alpha]_D^{24}$ = –74.6 (*c* = 0.53, methanol). ^{1}H NMR (300 MHz, CD_3OD) δ 7.46 (d, *J* = 8.8 Hz, 2H), 7.29 (s, 1H), 7.21 (s, 1H), 6.91 (d, *J* = 8.8 Hz, 2H), 5.57 (s, 1H), 5.17 (d, *J* = 7.5 Hz, 1H), 4.80 (m, 2H), 4.32 (dd, *J* = 9.7 and 4.3 Hz, 1H), 3.84- (m, 1H), 3.80 (s, 3H), 3.78-3.63 (complex m, 3H), 3.58 (m, 1H), 3.00 (m, 1H), 2.86 (dd, *J* = 17.7 and 5.0 Hz, 1H), 2.57 (dd, *J* = 17.7 and 10.4 Hz, 1H), 2.43 (s, 3H), 2.37 (m, 1H), 1.99 (m, 2H), 1.85 (s, 3H), 1.64 (m, 1H) (signals due to OH group protons not observed); ^{13}C NMR (75 MHz, $CDCl_3$) δ 179.3, 160.2, 157.3, 151.2, 148.0, 142.0, 140.3, 138.0, 129.9, 129.4,

127.7, 113.7, 110.2, 103.2, 101.9, 80.0, 77.3, 74.3, 73.5, 68.6, 67.0, 55.3, 40.5, 36.1, 27.1, 26.5, 20.8; IR ν_{max} 3360, 2918, 2849, 1646, 1591, 1541, 1471, 1207, 1075 cm^{-1}; HRMS (ESI, +ve) *m/z* (M+H)$^+$ calcd for $C_{29}H_{35}O_8$ 511.2326, found 511.2308.

Concentration of fraction B (t_R = 55 min) gave compound **33** (6.70 mg, 41%) as white crystals, m.p. = 122-123 °C, $[\alpha]_D^{24}$ = –50.2 (*c* = 0.52, dichloromethane); ^{1}H NMR (500 MHz, CD_2Cl_2) δ 7.41 (d, *J* = 8.8 Hz, 2H), 7.24 (s, 1H), 7.15 (s, 1H), 6.87 (d, *J* = 8.8 Hz, 2H), 5.50 (s, 1H), 4.89-4.71 (complex m, 3H), 4.30 (m, 1H), 3.89-3.80 (complex m, 2H), 3.79 (s, 3H), 3.71 (m, 1H), 3.58 (t, *J* = 9.3 Hz, 1H), 3.49 (m, 1H), 2.95 (m, 1H), 2.82 (m, 1H), 2.76 (m, 1H), 2.49 (m, 1H), 2.35 (s, 3H), 2.27 (m, 1H), 1.94 (m, 1H), 1.81 (s, 3H), 1.58 (m, 1H) (signals due to OH group protons not observed); ^{13}C NMR (126 MHz, CD_2Cl_2) δ 179.7, 160.6, 157.8, 151.7, 148.9, 142.5, 140.8, 138.0, 130.2, 129.9, 128.0, 113.8, 110.0, 103.8, 102.0, 80.5, 74.8, 73.8, 68.9, 67.4, 55.6, 41.0, 36.7, 36.5, 27.2, 27.0, 20.8; IR ν_{max} 3365, 2924, 1594, 1541, 1519, 1488, 1248, 1076, 1030, 831 cm^{-1}; HRMS (ESI, +ve) *m/z* (M+H)$^+$ calcd for $C_{29}H_{35}O_8$ 511.2326, found 511.2303.

Compound 5

A magnetically stirred solution of compound **33** (10.0 mg, 0.02 mmol) in acetic acid/water (1 mL of a 9:1 v/v mixture) was heated at 50 °C (oil-bath) for 10 min then cooled and concentrated under reduced pressure below 25 °C. The ensuing residue was subjected to flash chromatography (silica, 10:1 v/v ethyl acetate/methanol elution) to afford, after concentration of the appropriate fractions (R_f = 0.2 in 1:1 v/v petroleum ether/ethyl acetate), compound **5** (5 mg, 65%) as white crystals, m.p. = 81-82 °C $[\alpha]_D^{24}$ = –25.3 (*c* = 0.36, methanol). ^{1}H NMR (600 MHz, CD_3OD) δ 7.20 (s, 1H), 7.17 (s, 1H), 4.85 (m, 1H), 4.72 (m, 2H), 3.83 (m, 1H), 3.54 (m, 1H), 3.47-3.41 (complex m, 3H), 3.25 (m, 1H), 2.94 (m, 1H), 2.85 (m, 1H), 2.72 (broadened d, *J* = 17.9 Hz, 1H), 2.46 (m, 1H), 2.31 (s, 3H), 2.21 (m, 1H), 1.84 (m, 1H), 1.73 (s, 3H), 1.50 (complex m, 1H) (signals due to OH group protons not observed); ^{13}C NMR (150 MHz, CD_3OD) δ 178.5, 157.9, 152.3, 148.5, 142.3, 140.6, 135.7, 124.3, 108.9, 100.8, 77.5, 75.9, 73.1, 70.2, 61.3, 40.6, 36.2, 35.7, 26.6, 25.8, 19.5; IR ν_{max} 3346, 2923, 1592, 1533, 1483, 1399, 1247, 1104, 1071, 889 cm^{-1}; HRMS (ESI, +ve) *m/z* (M+H)$^+$ calcd for $C_{21}H_{29}O_7$ 393.1908, found 393.1909.

X-ray Crystallographic Studies

Crystallographic Data

Crystallographic Data for Compound ***(±)-20***

$C_{15}H_{21}BrO_2$, *M* = 313.22, *T* = 150 K, monoclinic, space group $P2_1/c$, *Z* = 4, *a* = 7.6303(8), *b* = 19.803(3), *c* = 9.3498(9) Å; β = 94.119(9)°; *V* = 1409.2(3) $Å^3$, D_x = 1.476g cm^{-3}, 4696 unique data ($2\theta_{max}$ = 58.866°), *R* = 0.0587 [for 2339 reflections with $I > 2.0\sigma(I)$]; *Rw* = 0.1470 (all data), *S* = 0.847.

Crystallographic Data for Compound ***33***

$C_{29}H_{34}O_8 \bullet CH_3OH$, *M* = 542.60, *T* = 170 K, orthorhombic, space group $P2_12_12_1$, *Z* = 4, *a* = 7.91563(16), *b* = 10.5994(3), *c* = 31.7457(7) Å; V = 2663.50(10) $Å^3$, D_x = 1.353 g cm^{-3}, 5241 unique data ($2\theta_{max}$ = 148.116°), *R* = 0.0424 [for 4884 reflections with $I > 2.0\sigma(I)$]; *Rw* = 0.0410 (all data), *S* = 1.038.

Structure Determinations

Diffraction data for compounds **(±)-20** and **33** were measured on a Bruker APEX-II CCD diffractometer (MoKα, graphite monochromator, λ = 0.71073 Å) Using OLEX2,[5] structures were solved by dual-space methods with the ShelXT[6] program and refined, using least squares minimization, with the ShelXL[7] package. Atomic coordinates, bond lengths and angles, and displacement parameters have been deposited at the Cambridge Crystallographic Data Centre (CCDC nos. 2091407 and 2388550). These data can be obtained free-of-charge via www.ccdc.cam.ac.uk/data_request/cif, by emailing data_request@ccdc.cam.ac.uk, or by contacting The Cambridge Crystallographic Data Centre, 12 Union Road, Cambridge CB2 1EZ, UK; fax: +44 1223 336033.

Biological Testing Protocols

Antibacterial Assays

The bacterium to be tested was streaked onto a Luria-Bertani (LB) for the susceptible strains and tryptic soy agar (TSA) for the multi-drug resistant pathogens and then incubated at 37 °C for 24 h. One colony was then transferred to fresh LB broth (4-5 mL) and the cell density was adjusted to 5-6 x 10^4-10^5 CFU/mL. Samples of compound **5** to be tested were dissolved in DMSO and diluted with water to give a 600 µM stock solution (20% DMSO) that was serially diluted with 20% DMSO to give concentrations ranging from 600 µM to 0.2 µM in 20% DMSO. An aliquot (10 µL) of each dilution was transferred to a 96-well microtiter plate and freshly prepared microbial broth (190 µL) was added to each well to give final concentrations of 30 – 0.01 µM in 1% DMSO. The plates were incubated at 37 °C for 24 h and the optical density of each well was measured spectrophotometrically at 600 nm using POLARstar Omega plate reader (BMG LABTECH, Offenburg, Germany). The test compound **5** was screened against Gram-positive *Staphylococcus aureus* ATCC 25923, Gram-negative *Escherichia coli* ATCC11775 as well as clinical isolates of extended-spectrum β-lactamase (ESBL)-resistant *Escherichia coli* (ESBL *E. coli* KUW-kw-AmH-11), methicillin-resistant *Staphylococcus aureus* (MRSA, AUS-RBWH-MRSA-02) and vancomycin-resistant *Enterococci* (VRE, AUS-RBWH-VRE-01). For the susceptible strains, the positive control was rifampicin (10 µM in 1% DMSO) and the negative control was 1% DMSO. For the MDR pathogens, the positive control was ampicillin for ESBL-resistant *Escherichia coli*, methicillin for MRSA and vancomycin for VRE. All studies were performed in duplicate from two independent cultures. MIC and IC_{50} values were calculated as the concentration of the compound or antibiotic required for 90% and 50% inhibition respectively of the bacterial cells using Prism 10.0 from GraphPad Software Inc. (La Jolla, CA).

Cytotoxicity Assays

The MTT assay was conducted using a slightly modified form of one described previously.[8] So, adherent SW620 (susceptible human colorectal carcinoma) were cultured in Roswell Park Memorial Institute (RPMI) 1640 medium. All cells were cultured as adherent mono-layers in flasks supplemented with 10% foetal bovine serum, L-glutamine (2 mM), penicillin (100 unit/mL) and streptomycin (100 µg/mL), in a humidified 37 °C

incubator supplied with 5% CO_2. Cells were then harvested with trypsin and dispensed into 96-well microtiter assay plates at 3,000 cells/well and after which they were incubated for 18 h at 37 °C with 5% CO_2 (so as to allow cells to attach as adherent mono-layers). Test compound **5** was dissolved in 20% DMSO in PBS (v/v) and aliquots (10 μL) applied to cells over a series of final concentrations ranging from 10 nM to 30 μM. After 48 h of incubation at 37 °C with 5% CO_2 an aliquot (10 μL) of 3-(4,5-dimethylthiazol-2-yl)-2,5-diphenyltetrazolium bromide (MTT) in phosphate buffered saline (PBS, 5 mg/mL) was added to each well (final concentration 0.5 mg/mL) and the microtiter plates were incubated for a further 4 h at 37 °C with 5% CO_2. After final incubation, the medium was aspirated and precipitated formazan crystals dissolved in DMSO (100 μL/well). The absorbance of each well was measured at 600 nm with a POLARstar Omega plate reader (BMG LABTECH, Offenburg, Germany). Where relevant, IC_{50} values were calculated using Prism 10.0, as the concentration of analyte required for 50% inhibition of cancer cell growth (compared to negative controls). Negative control was 1% aqueous DMSO, while positive control was doxorubicin (final concentration 30 μM). All experiments were performed in duplicate from two independent cell cultures.

Antifungal Assays

Sabouraud Dextrose (SD) agar plates inoculated with *Candida albicans* ATCC10231 were incubated at 27 °C for 48 h and after which several colonies were transferred to fresh sterile SD broth (4 mL) which was incubated at 27 °C for 48 h and, following the measurement of optical density, the cell density was adjusted to 5×10^5 CFU/mL. An aliquot (10 μL) of analytes as prepared above for the antibacterial assays was transferred to a 96-well microtiter plate and freshly prepared fungal broth (190 μL) was added to each well to give final concentrations of 30–0.01 μM in 1% DMSO. The resulting assay plates were incubated at 27 °C for 48 h and the optical density of each well was measured spectrophotometrically at 600 nm using the POLARstar Omega plate reader (BMG LABTECH, Offenburg, Germany). The positive control was amphotericin (10 μM in 1% DMSO) and the negative control was 1% DMSO together with the SD broth medium lacking the fungal innoculant. Each analysis was repeated twice.

References

[1]. W. C. Still, M. Kahn, A. Mitra, *J. Org. Chem*. **1978**, *43*, 2923-2925.

[2]. A. B. Pangborn, M. A. Giardello, R. H. Grubbs, R. K. Rosen, F. J. Timmers, *Organometallics* **1996**, *15*, 1518-1520.

[3]. Q. Chen, M. G. Banwell, M. G. Gardiner, P. Lan, S. Tan, *J. Org. Chem*. **2024**, *89*, 13530-13539.

[4]. Q. Chen, P. Lan, S. Tan, M. G. Banwell, *Org. Lett*. **2023**, *25*, 384-388.

[5]. O. V. Dolomanov, L. J. Bourhis, R. J. Gildea, J. A. K. Howard, H. Puschmann, *J. Appl. Cryst*. **2009**, *42*, 339-341.

[6]. G. M. Sheldrick, *Acta Cryst*. **2015**, *A71*, 3-8.

[7]. G. M. Sheldrick, *Acta Cryst*. **2015**, *C71*, 3-8.

[8]. Y. Y. Han, W. Yang, P. Lan, Z. G. Khalil, R. J. Capon, M. G. Banwell, *J. Nat. Prod*. **2024**, *87*, 2310-2316.

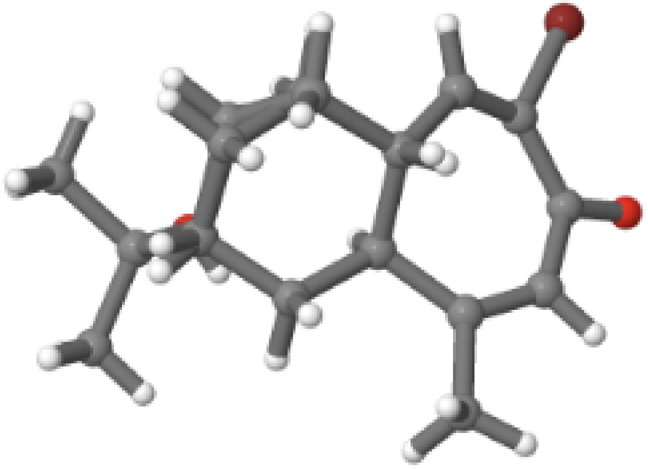

Figure S1: Plot derived from the single-crystal X-ray analysis of compound (±)-**20** showing disorder in the six-membered ring (crystal grown from 2:1 v/v petroleum ether/ethyl acetate at 4°C) (CCDC 2091407)

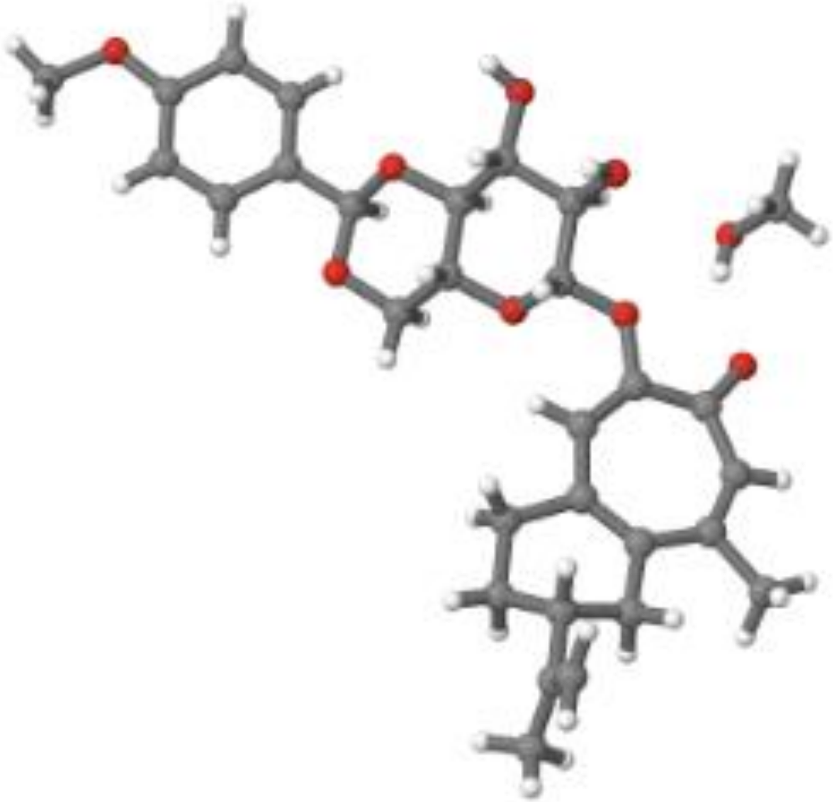

Figure S2: Plot derived from the single-crystal X-ray analysis of the methanolic solvate of compound **33** (crystal grown from 10:1 v/v dichloromethane/methanol at 4°C) (CCDC 2388550)

300 MHz ^{1}H NMR Spectrum of Compound (±)-**15**
(recorded in $CDCl_3$)

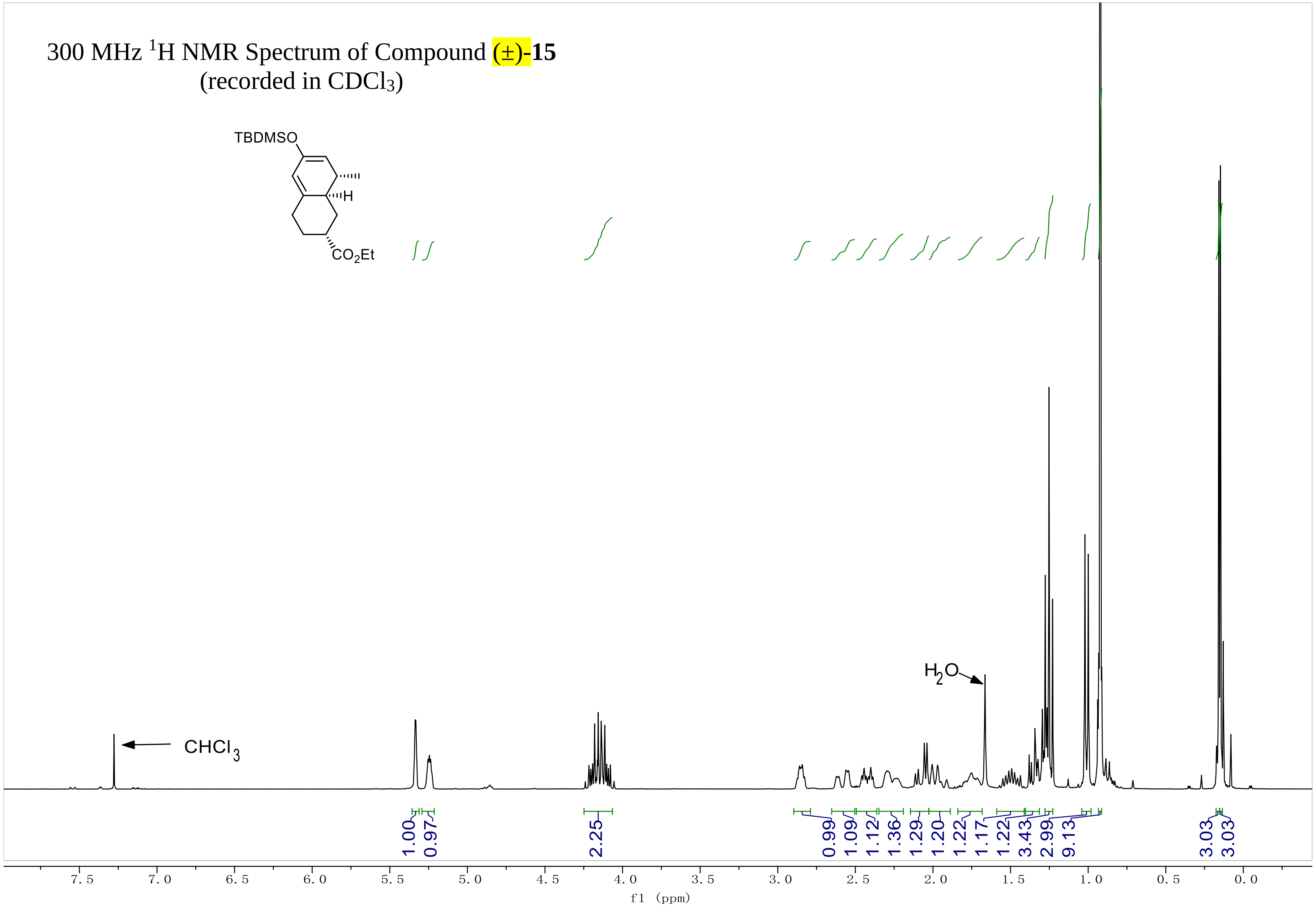

75 MHz $^{13}C\{^{1}H\}$ NMR Spectrum of Compound (±)-**15**
(recorded in $CDCl_3$)

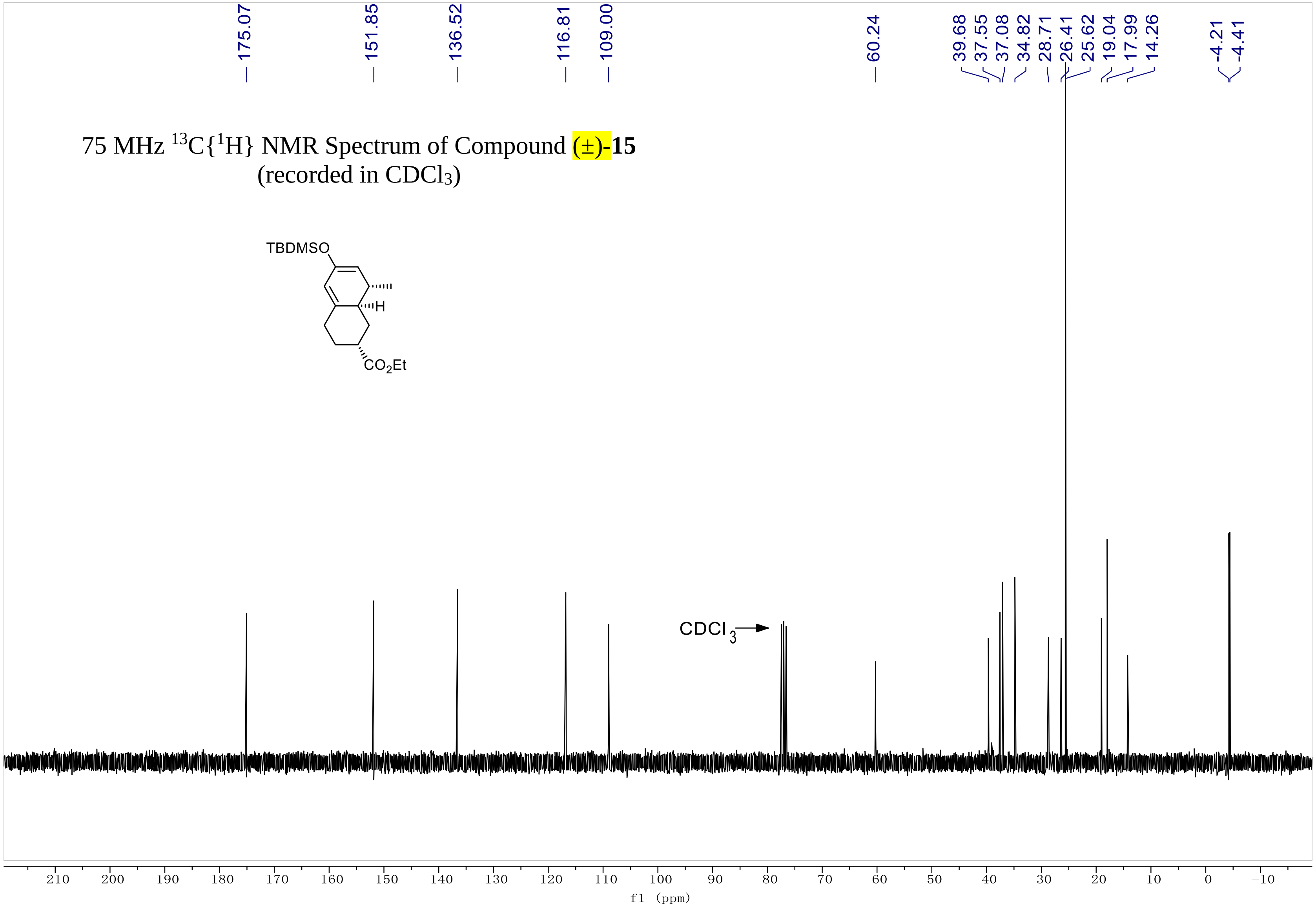

300 MHz ^{1}H NMR Spectrum of Compound (±)-**16**
(recorded in $CDCl_3$)

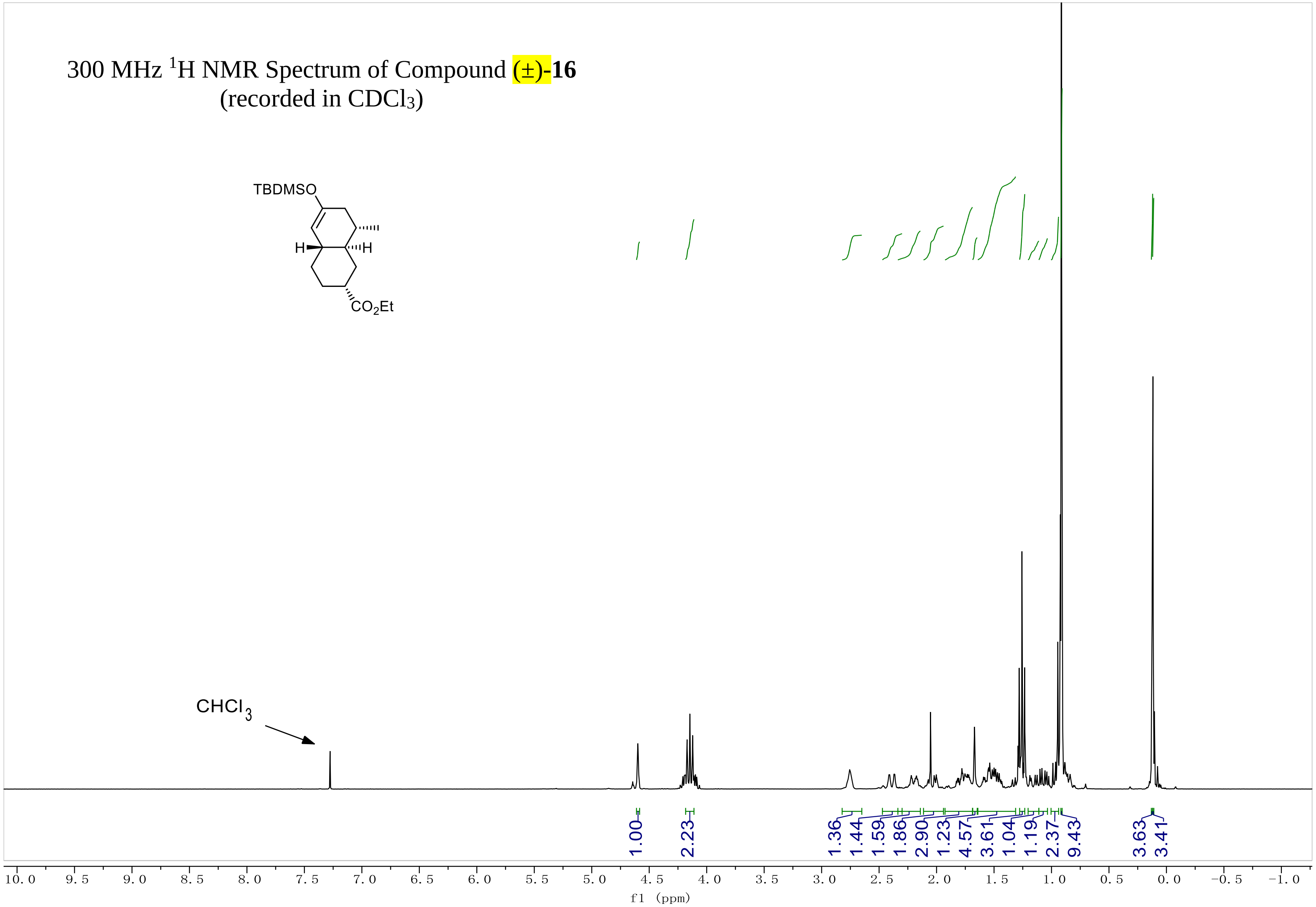

75 MHz $^{13}C\{^{1}H\}$ NMR Spectrum of Compound (±)-**16**
(recorded in $CDCl_3$)

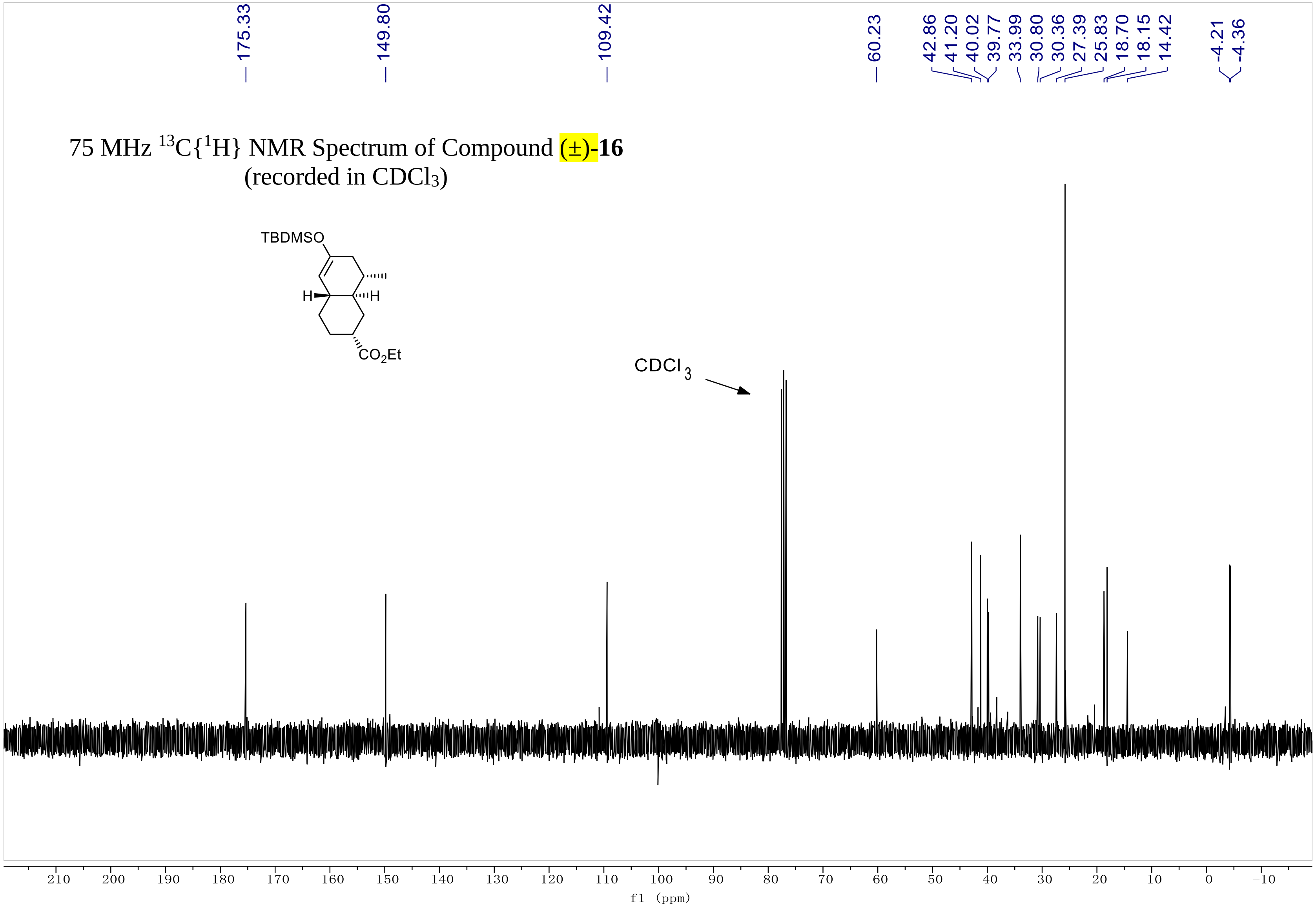

300 MHz ^{1}H NMR Spectrum of Compound **(±)-17**
(recorded in $CDCl_3$)

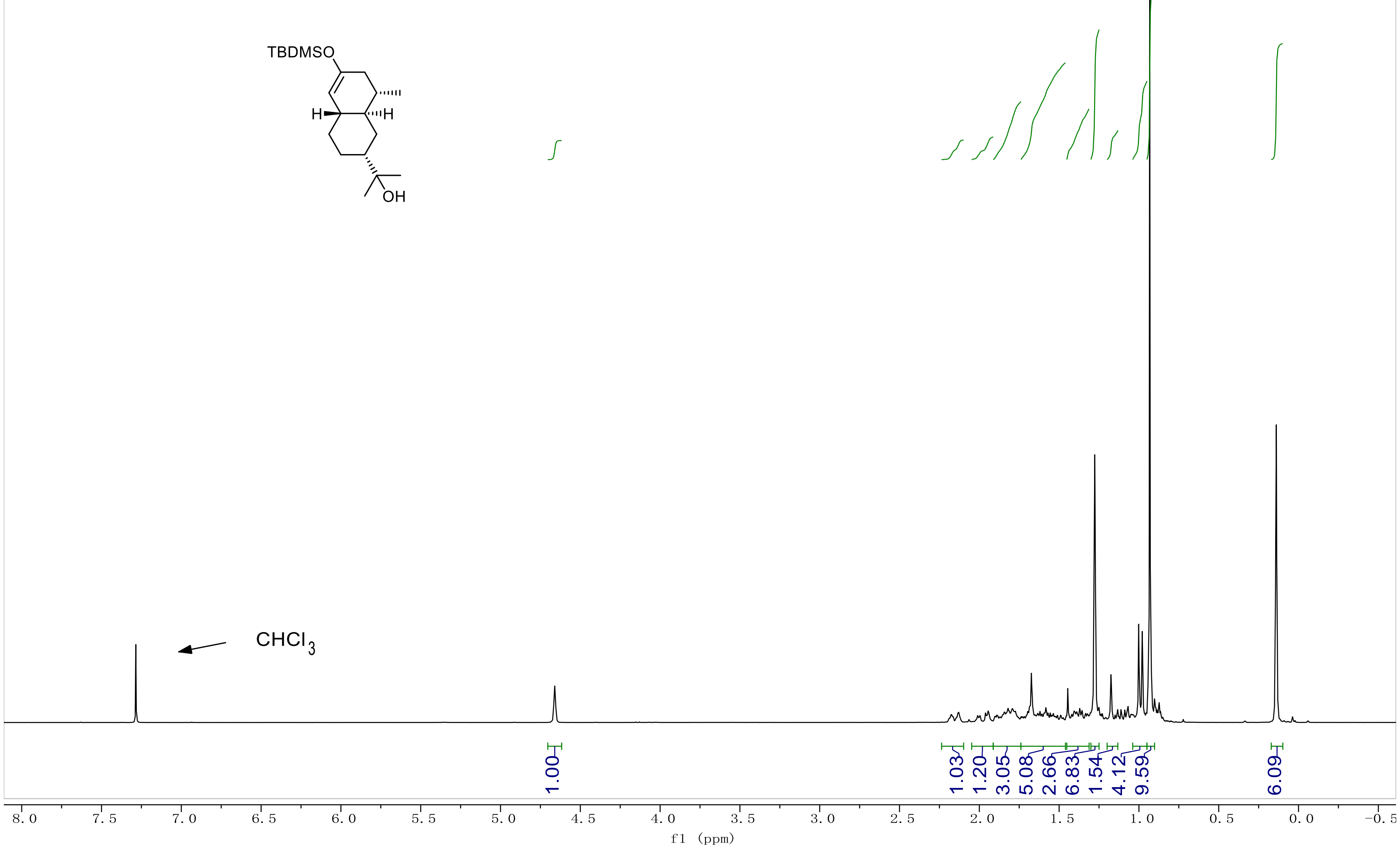

75 MHz $^{13}C\{^{1}H\}$ NMR Spectrum of Compound (±)-**17**
(recorded in $CDCl_3$)

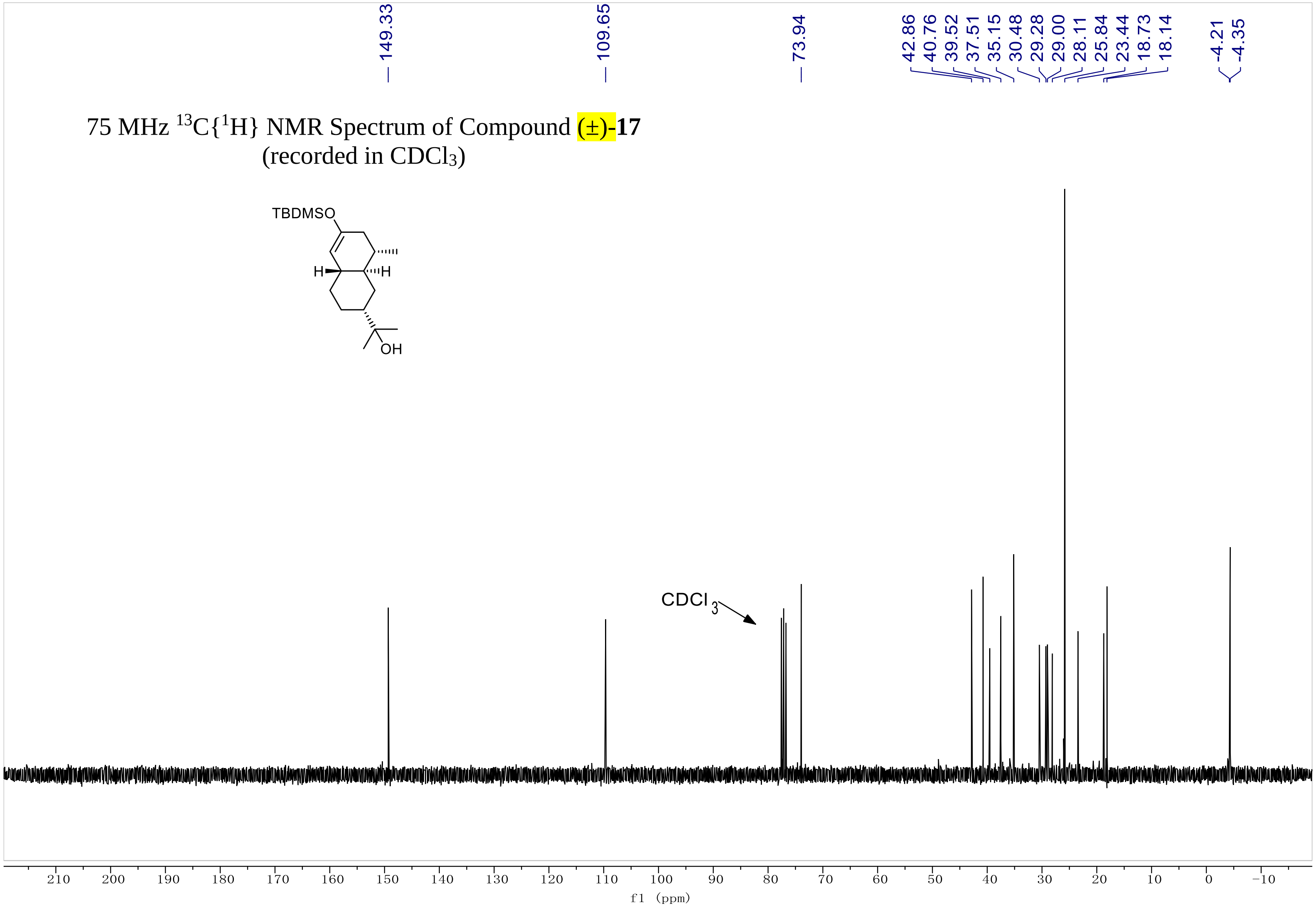

300 MHz ^{1}H NMR Spectrum of Compound (±)-**19**
(recorded in $CDCl_3$)

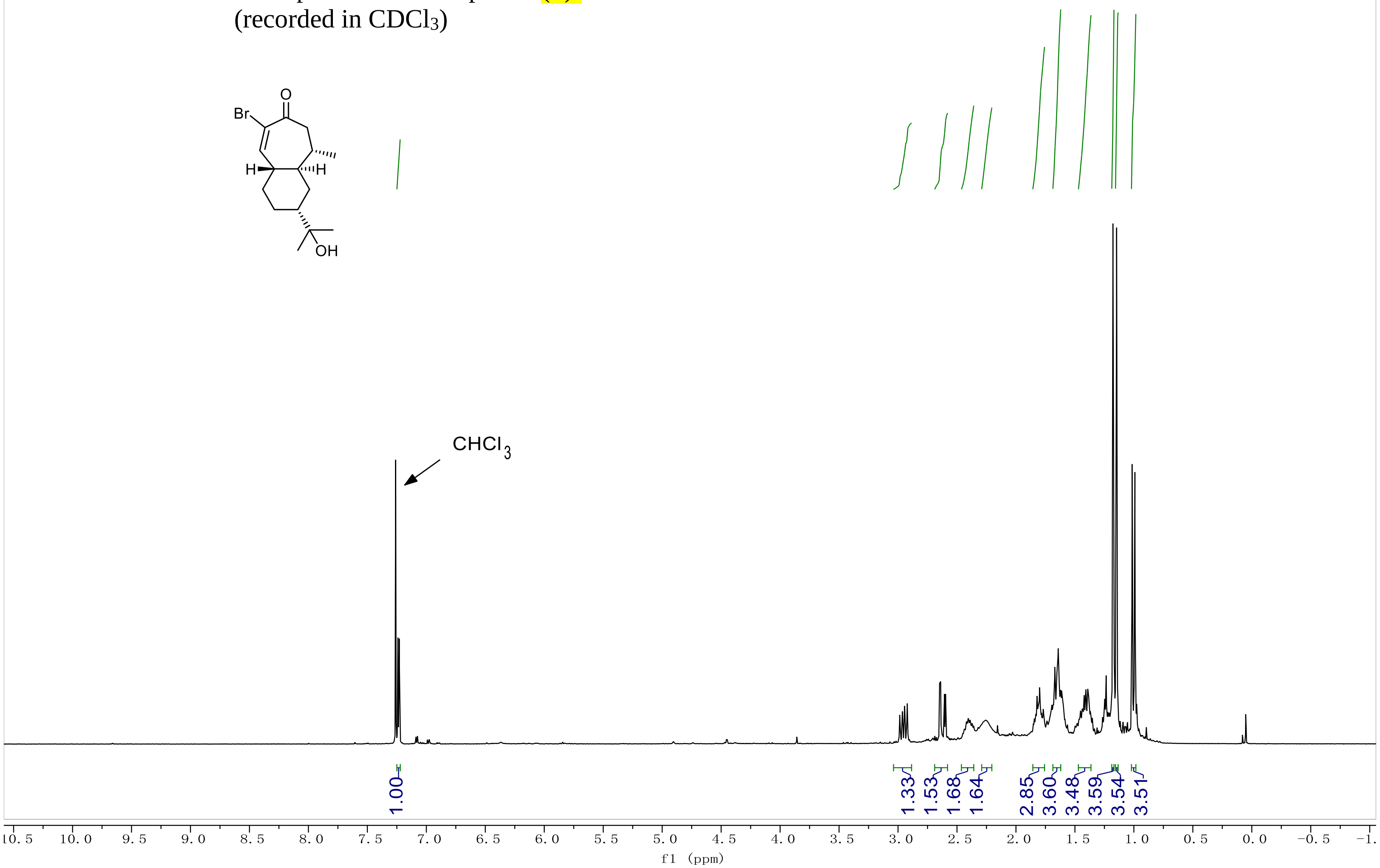

75 MHz $^{13}C\{^{1}H\}$ NMR Spectrum of Compound (±)-**19**
(recorded in $CDCl_3$)

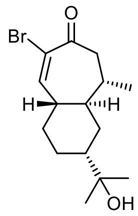

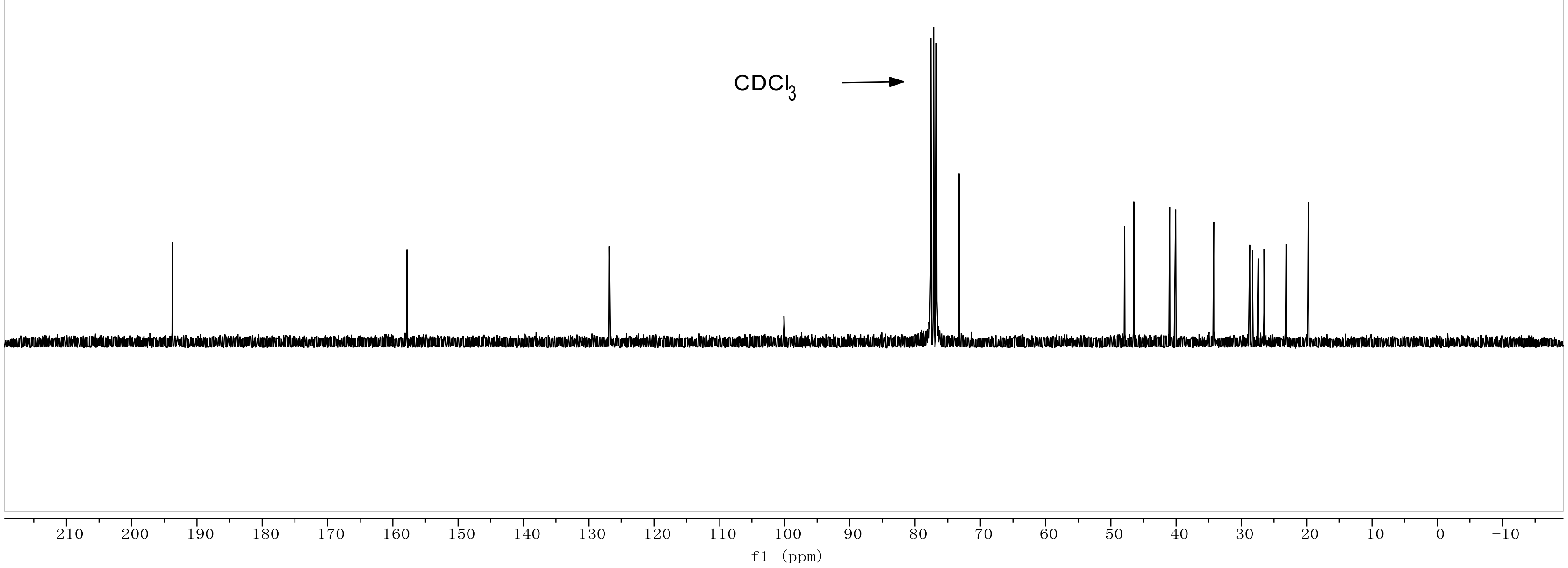

300 MHz ^{1}H NMR Spectrum of Compound (±)-**20**
(recorded in $CDCl_3$)

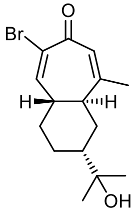

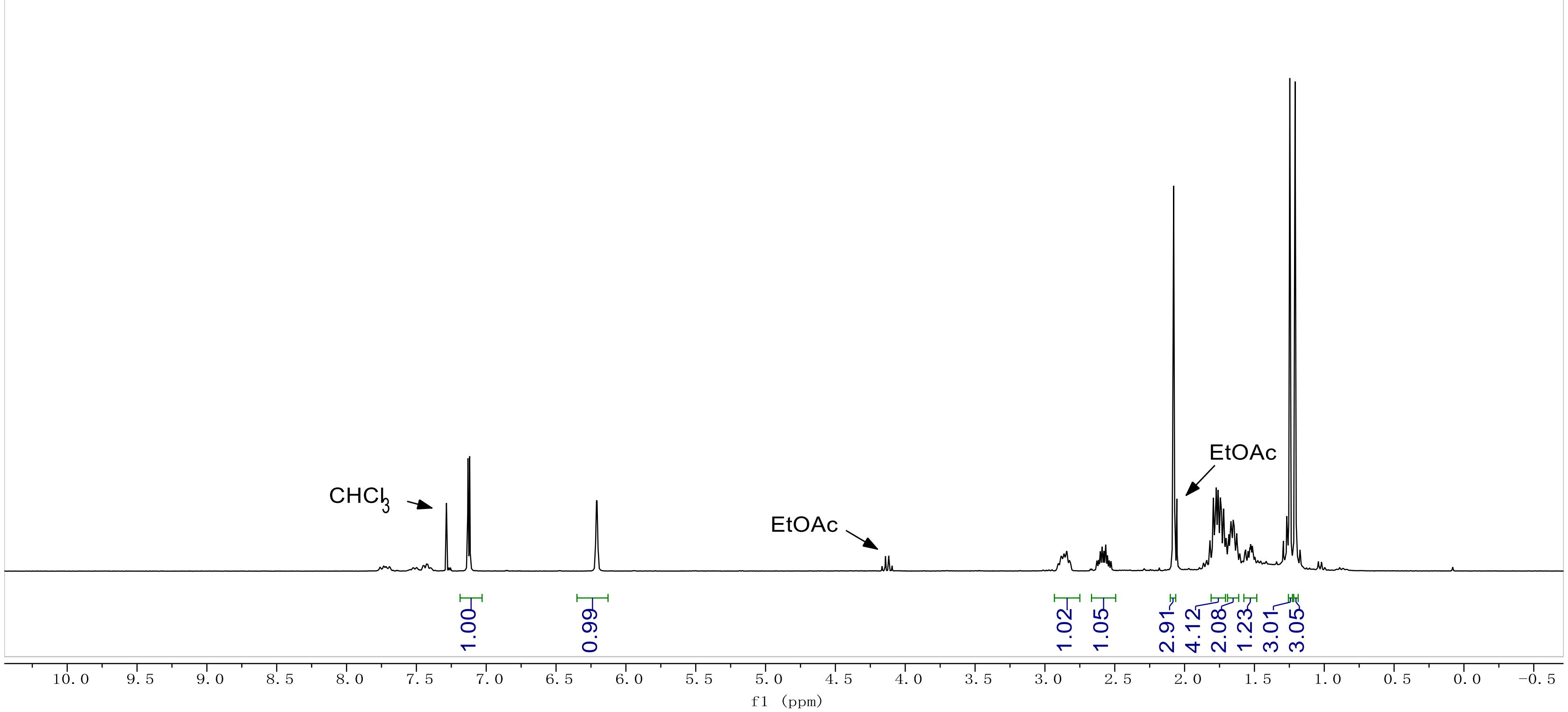

75 MHz $^{13}C\{^{1}H\}$ NMR Spectrum of Compound (±)-**20**
(recorded in $CDCl_3$)

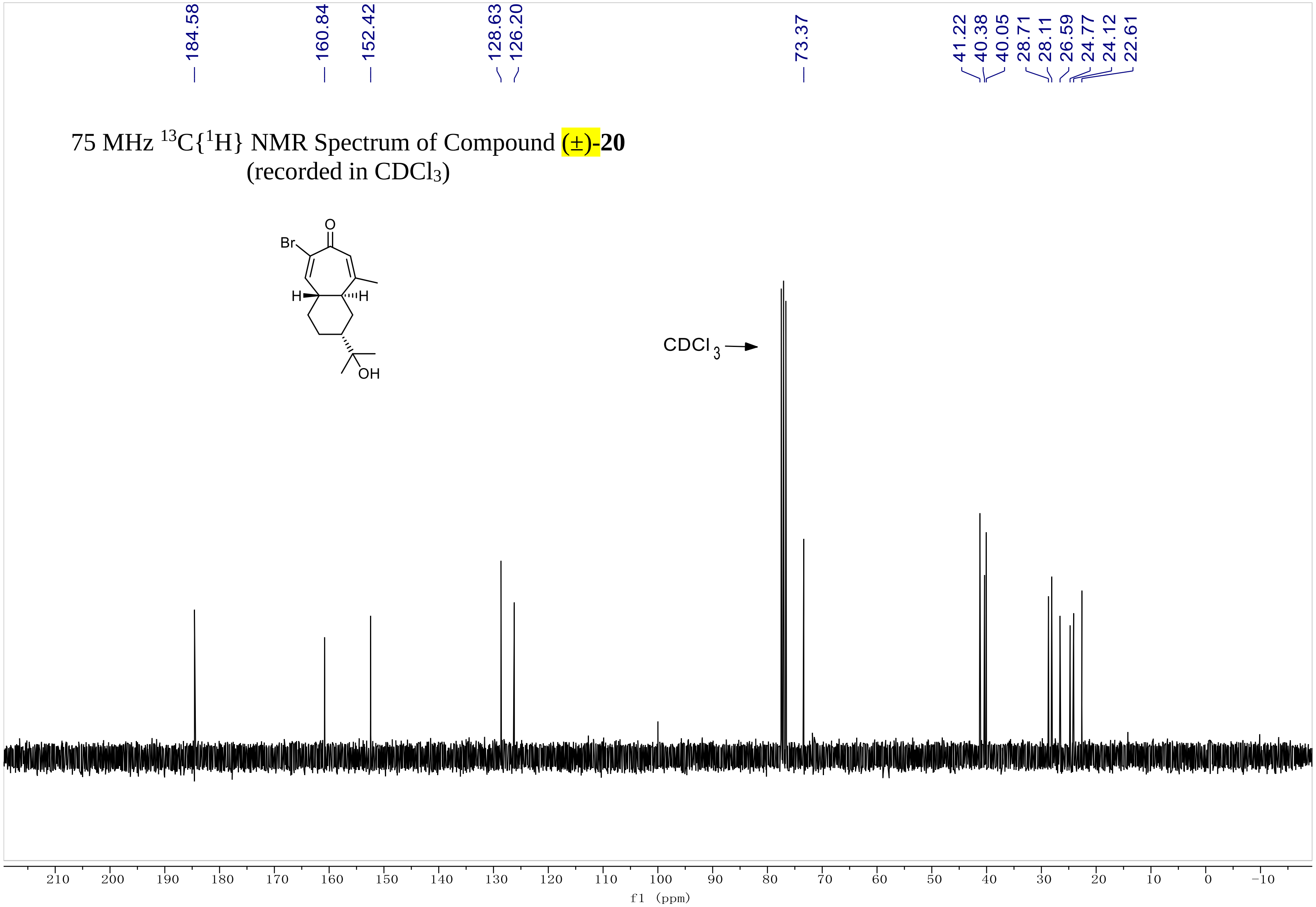

300 MHz ^1H NMR Spectrum of Compound (±)-**7**
(recorded in $CDCl_3$)

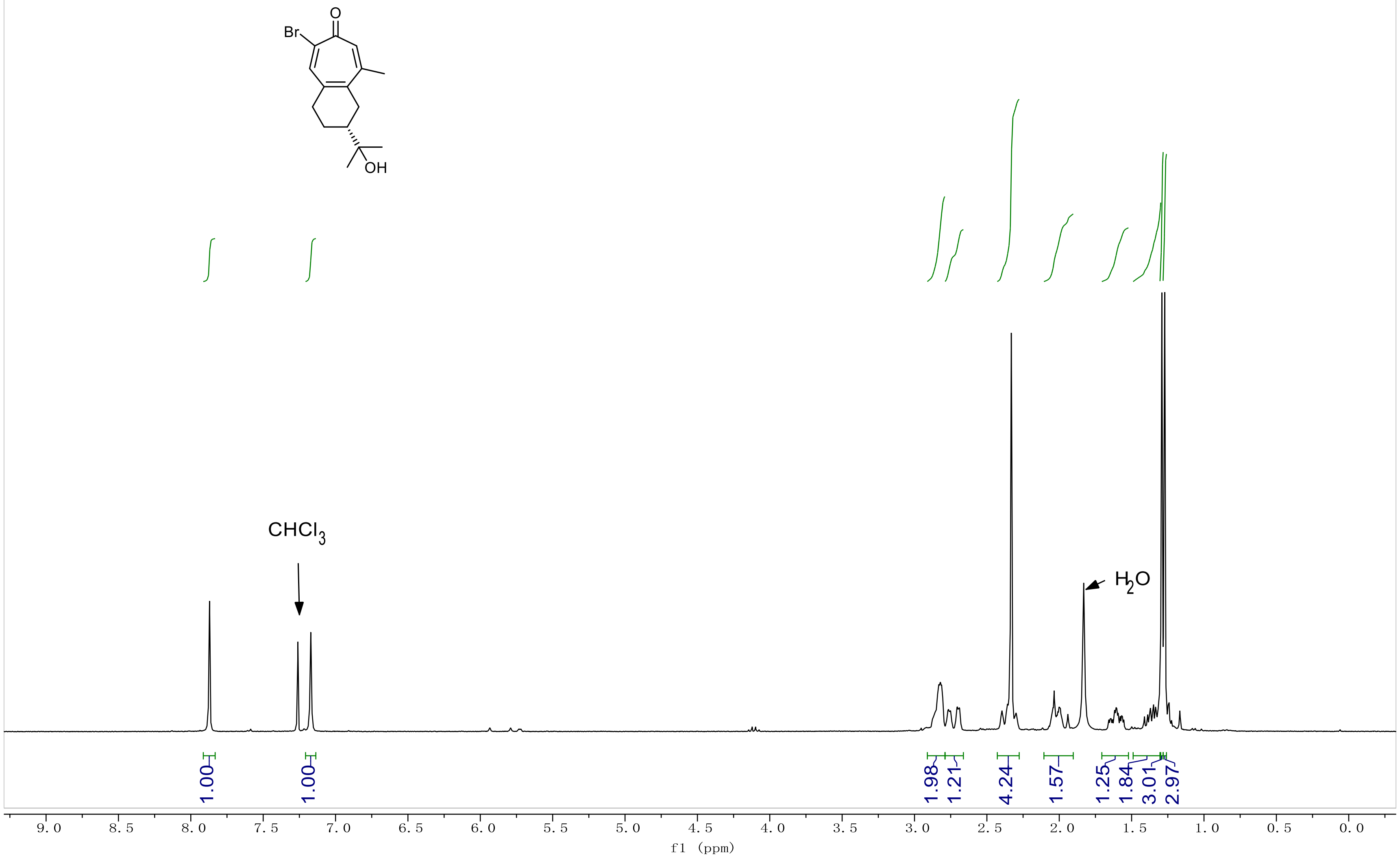

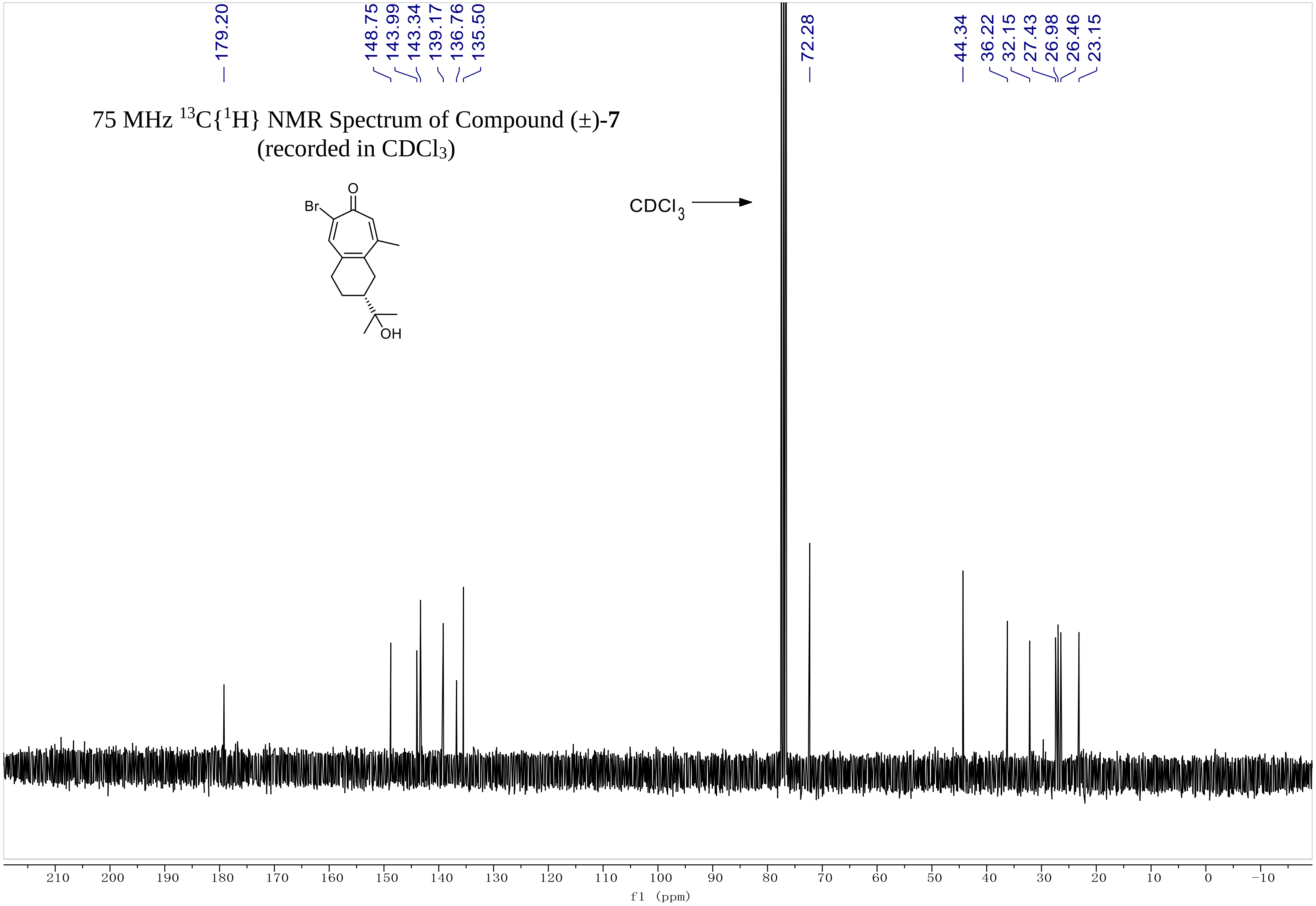
179.20
148.75
143.99
143.34
139.17
136.76
135.50
72.28
44.34
36.22
32.15
27.43
26.98
26.46
23.15
75 MHz $^{13}C\{^{1}H\}$ NMR Spectrum of Compound (±)-**7**
(recorded in $CDCl_3$)
O
Br
OH
$CDCl_3$
210
200
190
180
170
160
150
140
130
120
110
100
90
80
70
60
50
40
30
20
10
0
−10
f1 (ppm)

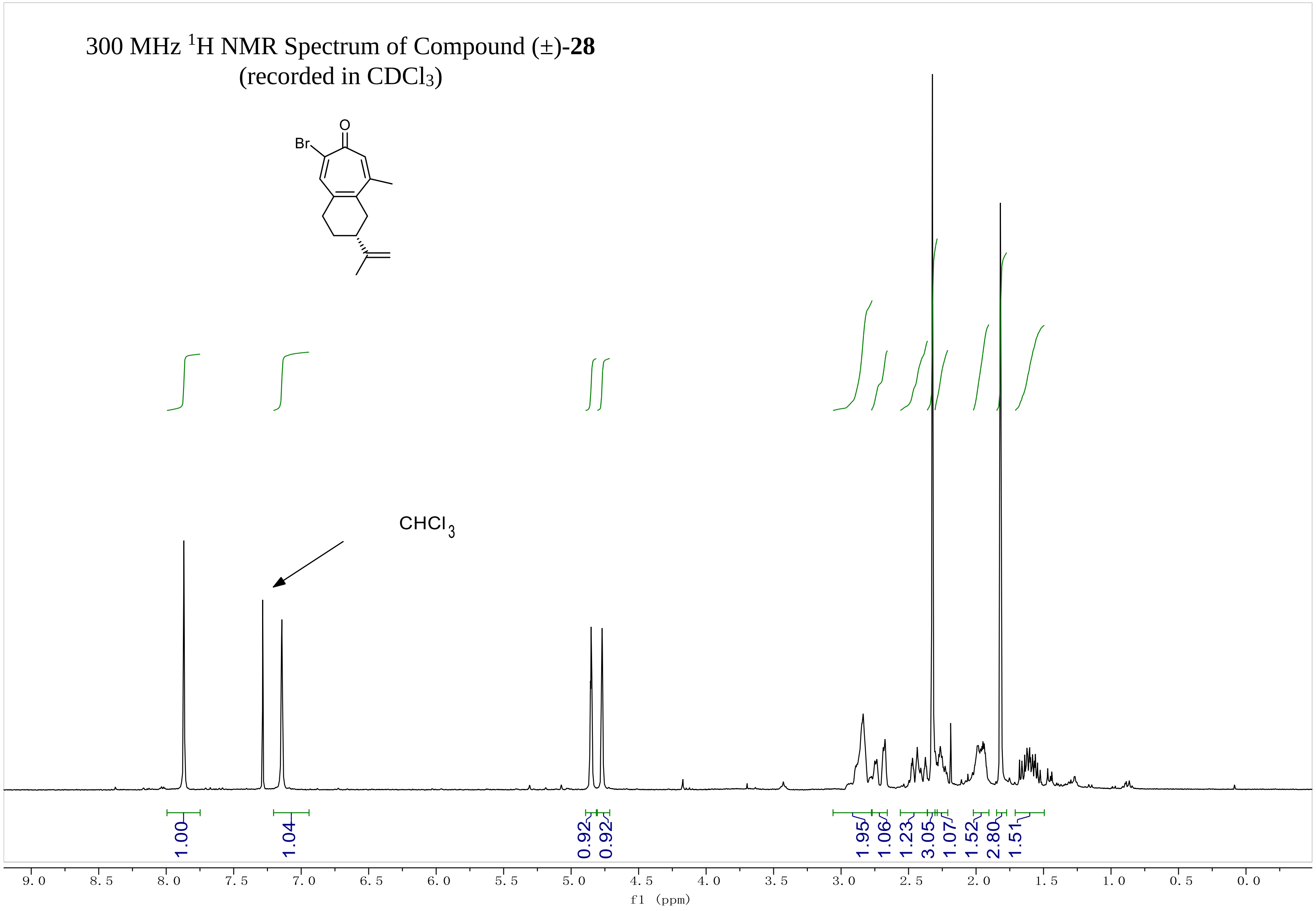
300 MHz ^{1}H NMR Spectrum of Compound (±)-**28**
(recorded in $CDCl_3$)
Br
O
$CHCl_3$
1.00
1.04
0.92
0.92
1.95
1.06
1.23
3.05
1.07
1.52
2.80
1.51
9.0 8.5 8.0 7.5 7.0 6.5 6.0 5.5 5.0 4.5 4.0 3.5 3.0 2.5 2.0 1.5 1.0 0.5 0.0
f1 (ppm)

75 MHz $^{13}C\{^{1}H\}$ NMR Spectrum of Compound (±)- **28**
[recorded in $(CD_3)_2SO$]

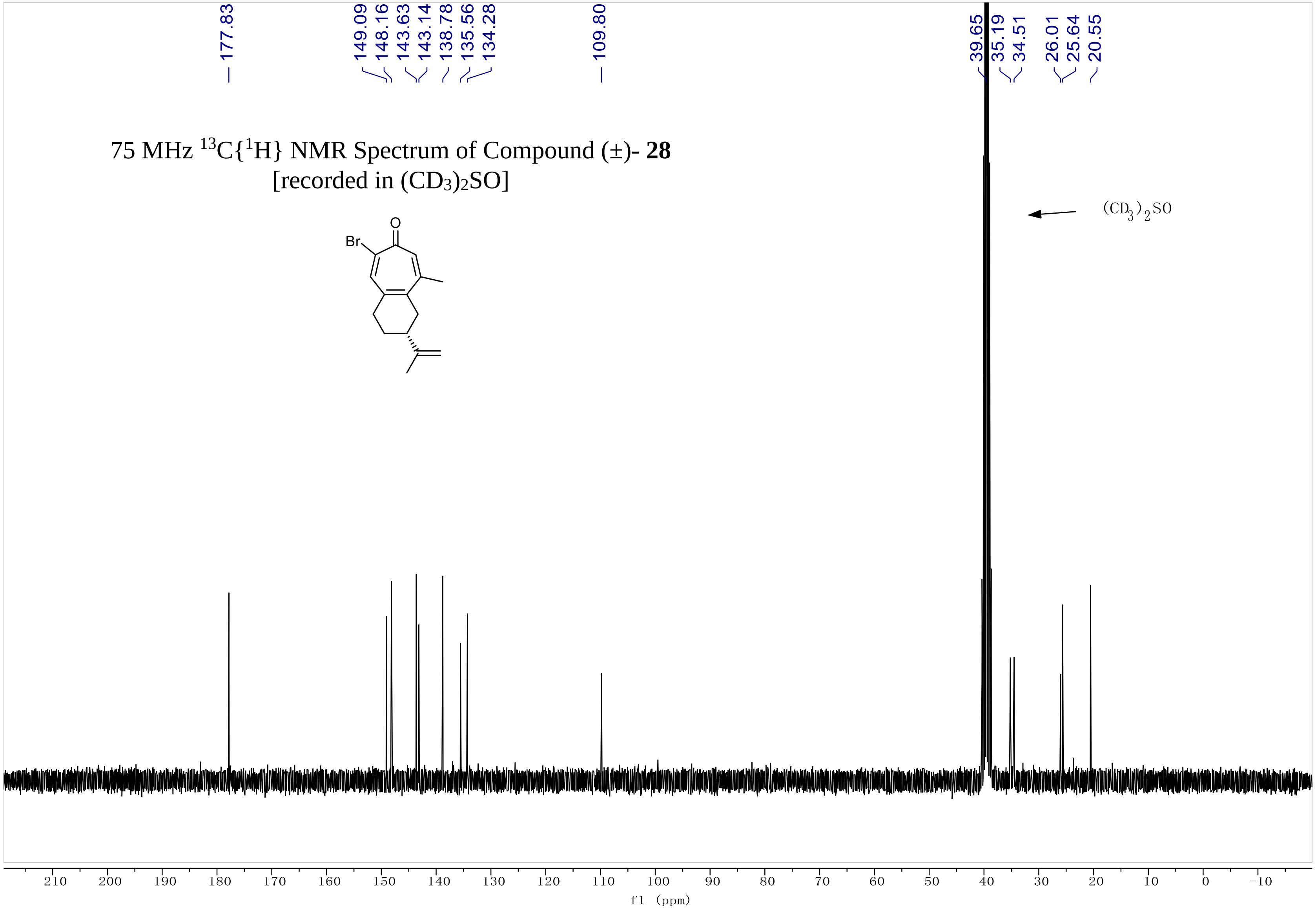

300 MHz ^{1}H NMR Spectrum of Compound **30**
(recorded in $CDCl_3$)

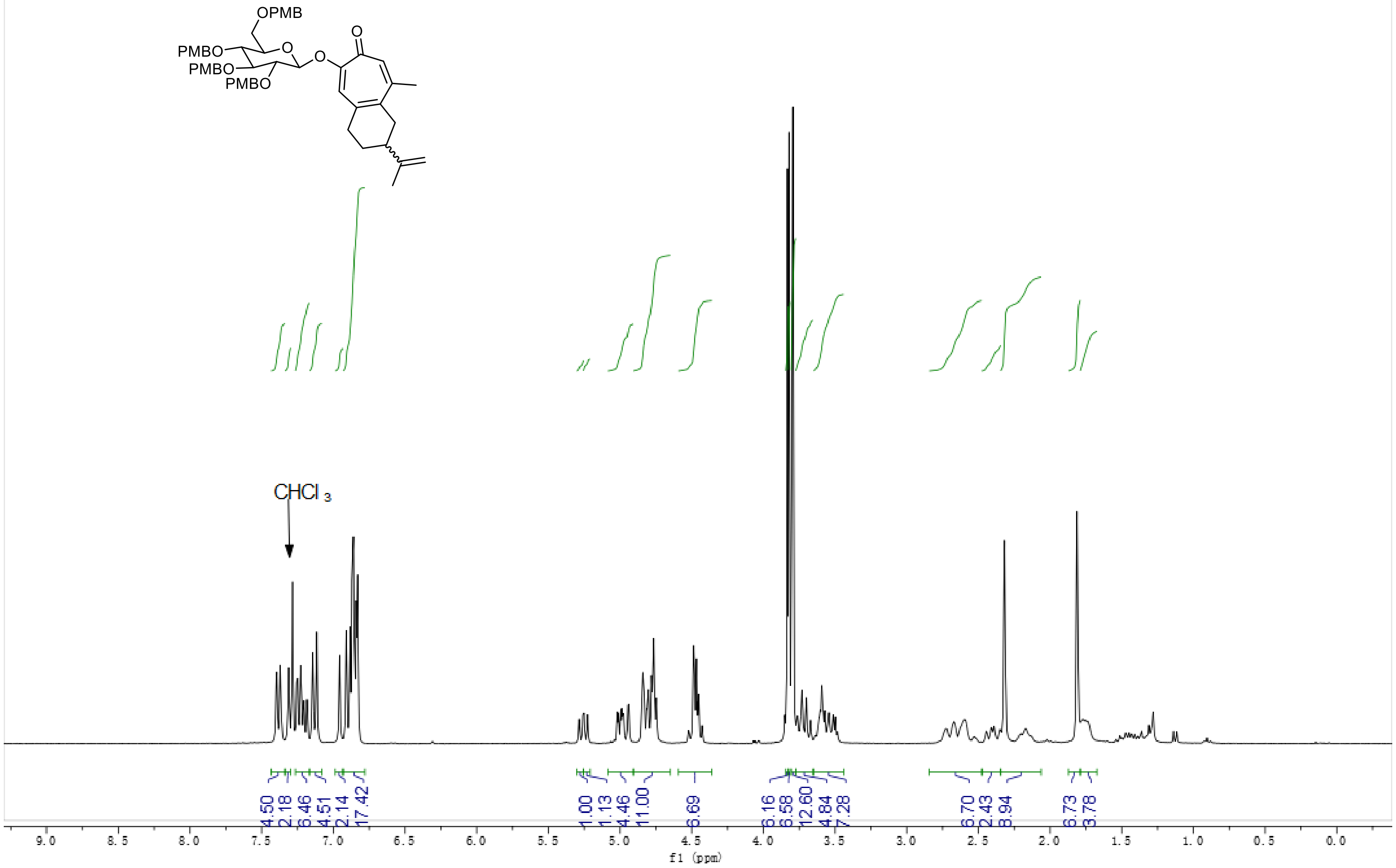

75 MHz $^{13}C\{^{1}H\}$ NMR Spectrum of Compound **30**
(recorded in $CDCl_3$)

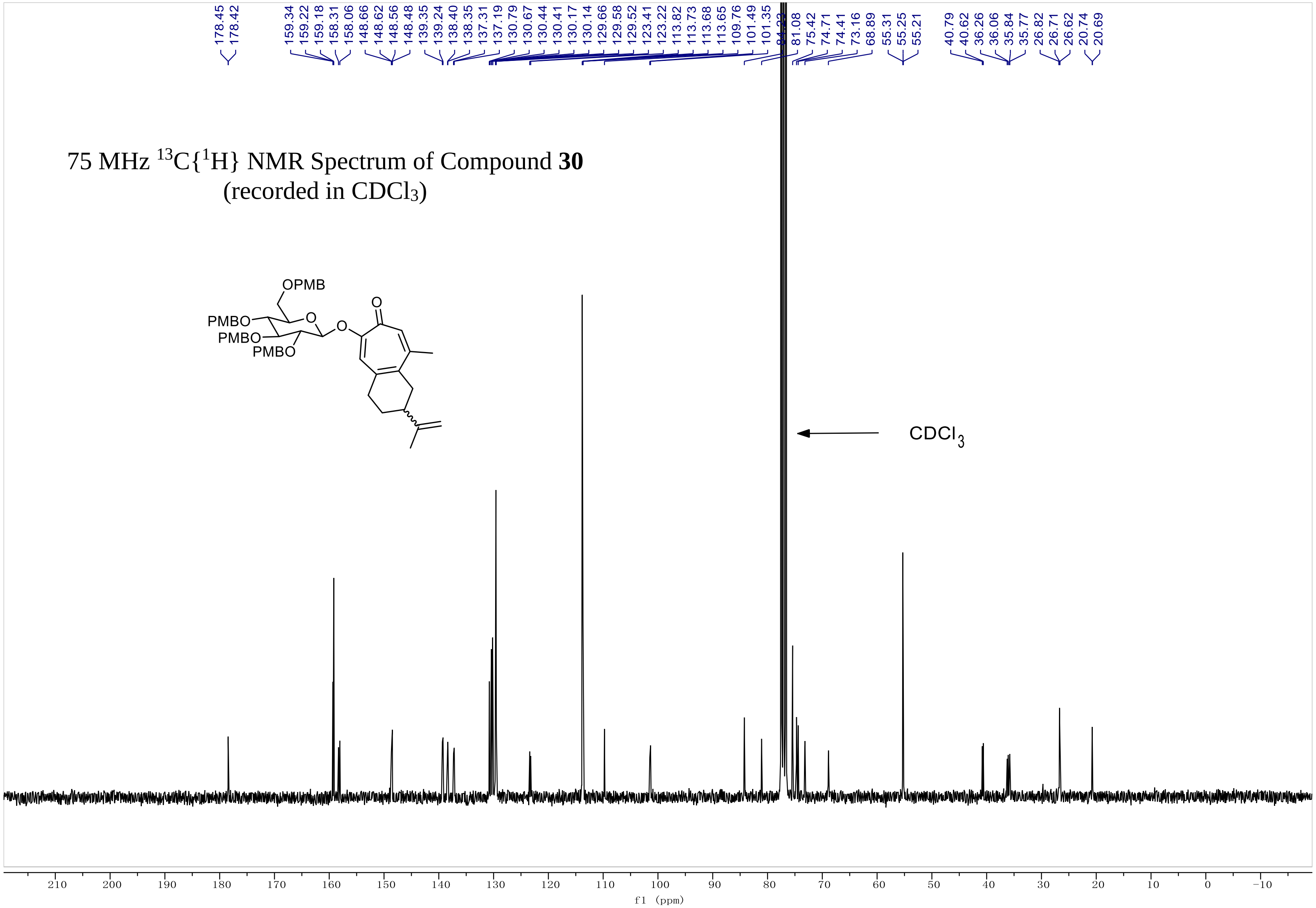

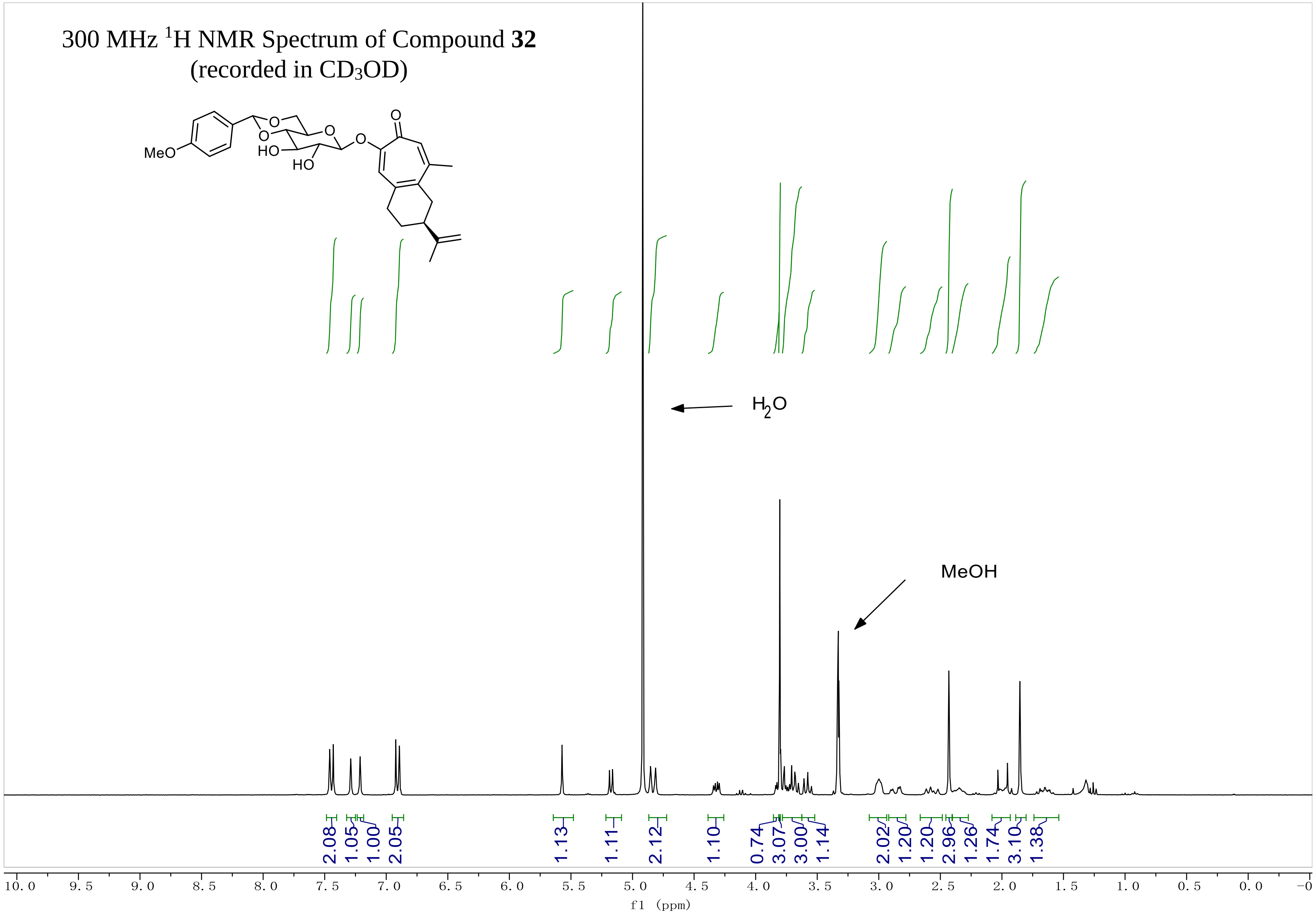
300 MHz ¹H NMR Spectrum of Compound **32**
(recorded in CD_3OD)
MeO
HO
HO
O
H_2O
MeOH
2.08
1.05
1.00
2.05
1.13
1.11
2.12
1.10
0.74
3.07
3.00
1.14
2.02
1.20
1.20
2.96
1.26
1.74
3.10
1.38
10.0
9.5
9.0
8.5
8.0
7.5
7.0
6.5
6.0
5.5
5.0
4.5
4.0
3.5
3.0
2.5
2.0
1.5
1.0
0.5
0.0
f1 (ppm)

75 MHz $^{13}C\{^1H\}$ NMR Spectrum of Compound **32**
(recorded in $CDCl_3$)

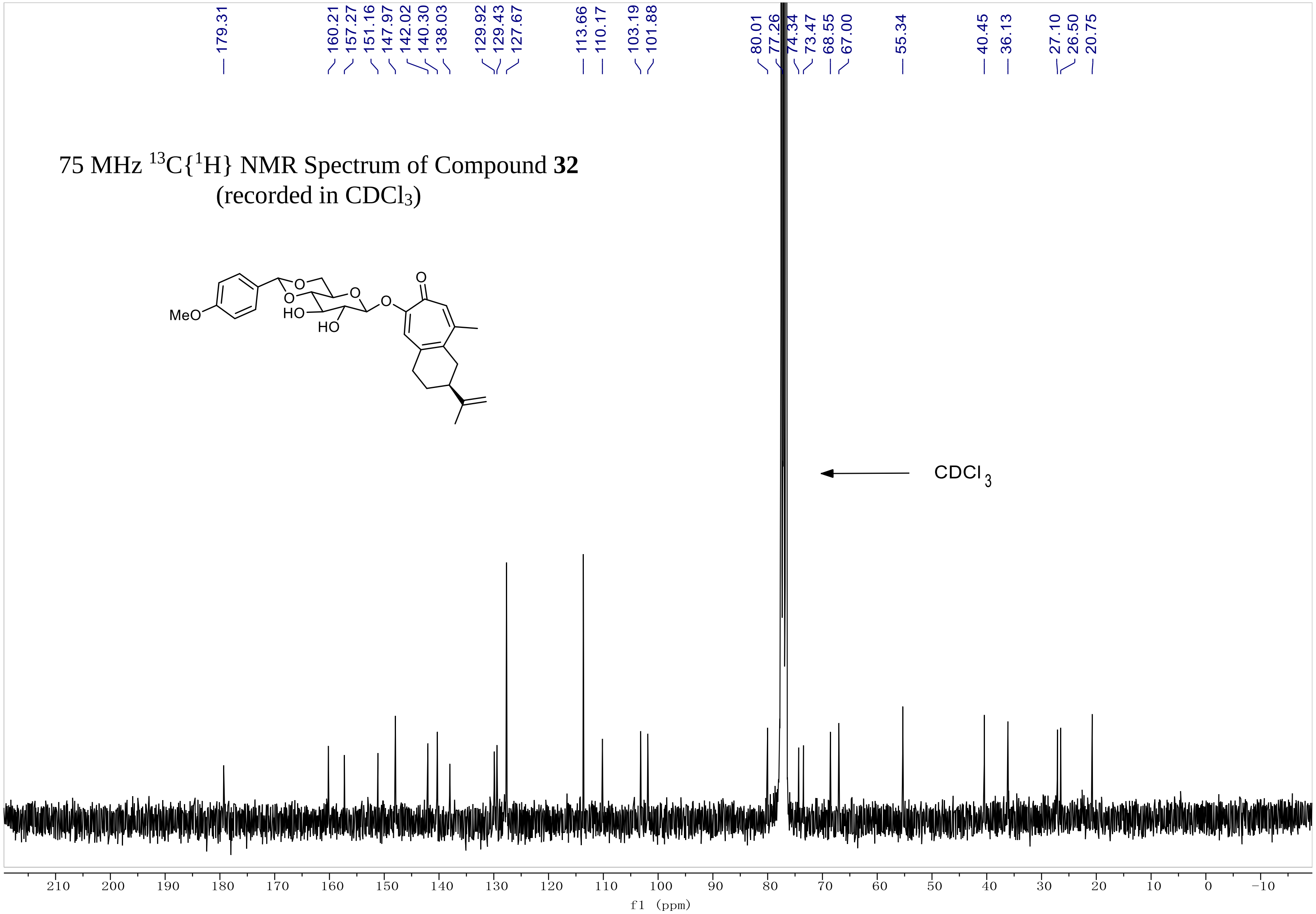

300 MHz ^{1}H NMR Spectrum of Compound **33**
(recorded in CD_2Cl_2)

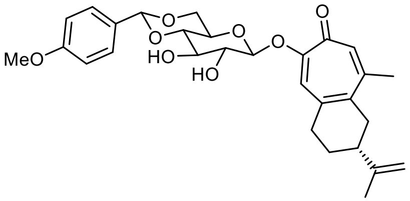

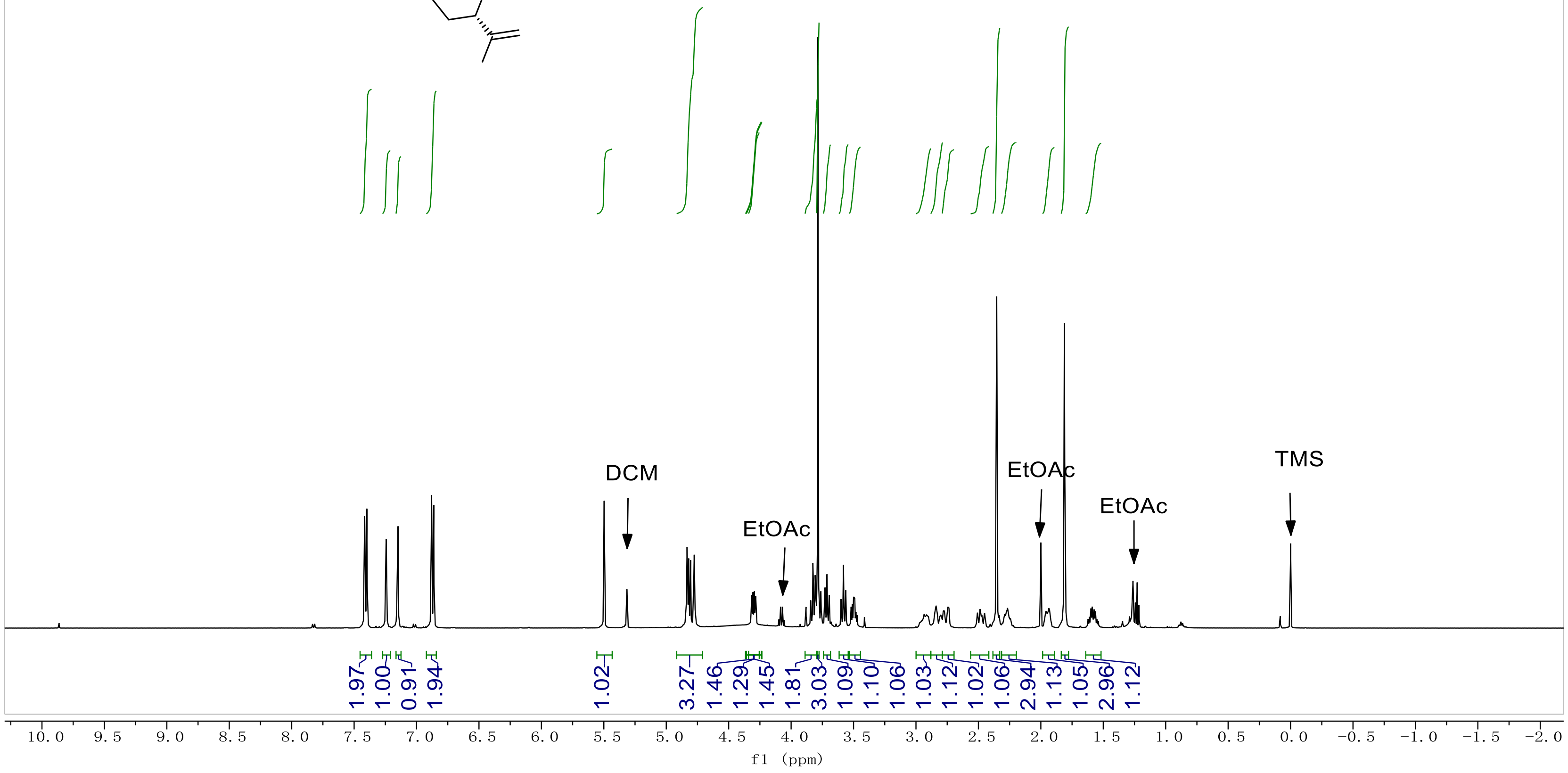

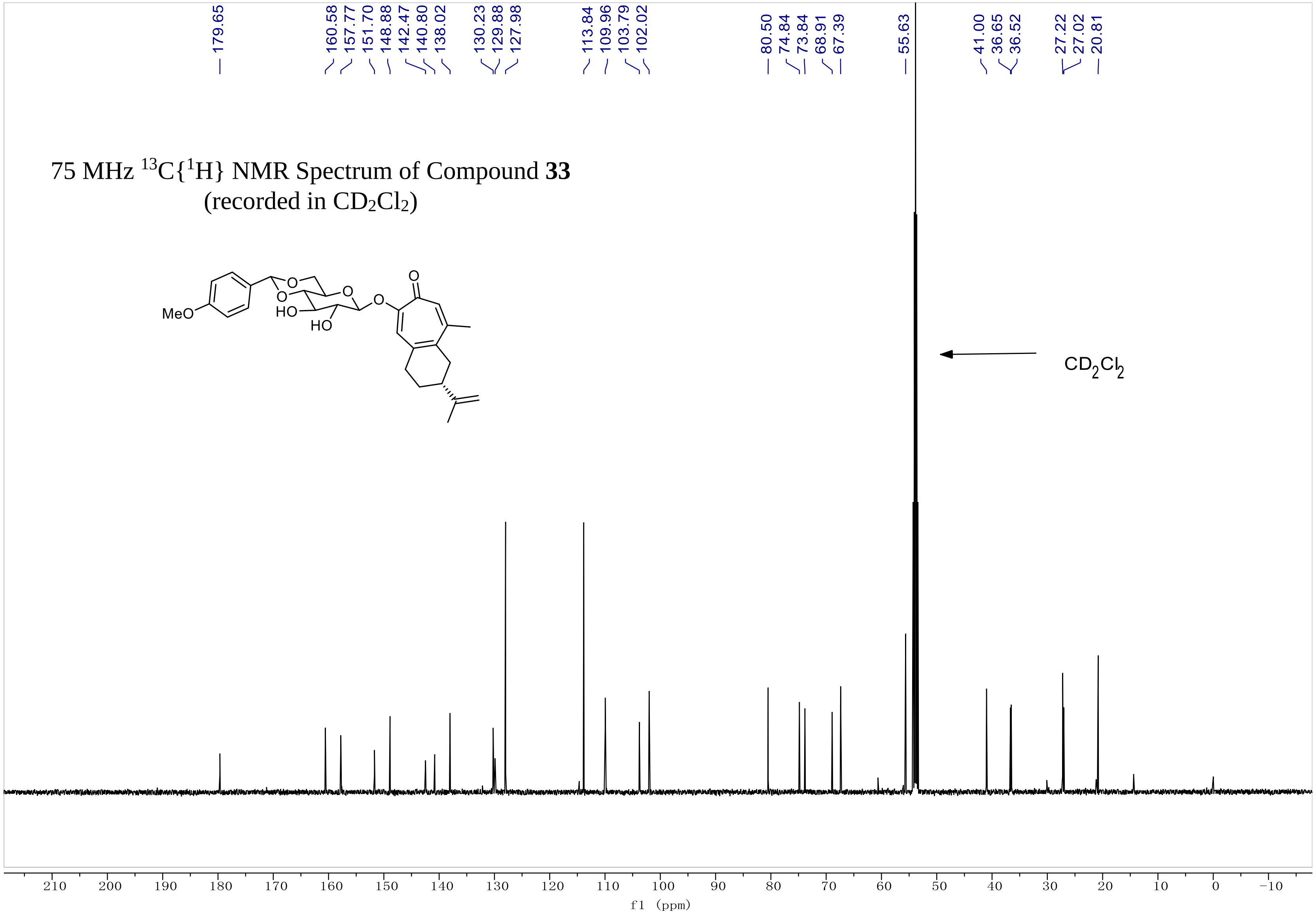
75 MHz $^{13}C\{^{1}H\}$ NMR Spectrum of Compound **33**
(recorded in CD_2Cl_2)
179.65
160.58
157.77
151.70
148.88
142.47
140.80
138.02
130.23
129.88
127.98
113.84
109.96
103.79
102.02
80.50
74.84
73.84
68.91
67.39
55.63
41.00
36.65
36.52
27.22
27.02
20.81
MeO
O
O
O
O
HO
HO
O
CD_2Cl_2
210 200 190 180 170 160 150 140 130 120 110 100 90 80 70 60 50 40 30 20 10 0 −10
f1 (ppm)

600 MHz ¹H NMR Spectrum of Compound **5**
(recorded in CD_3OD)

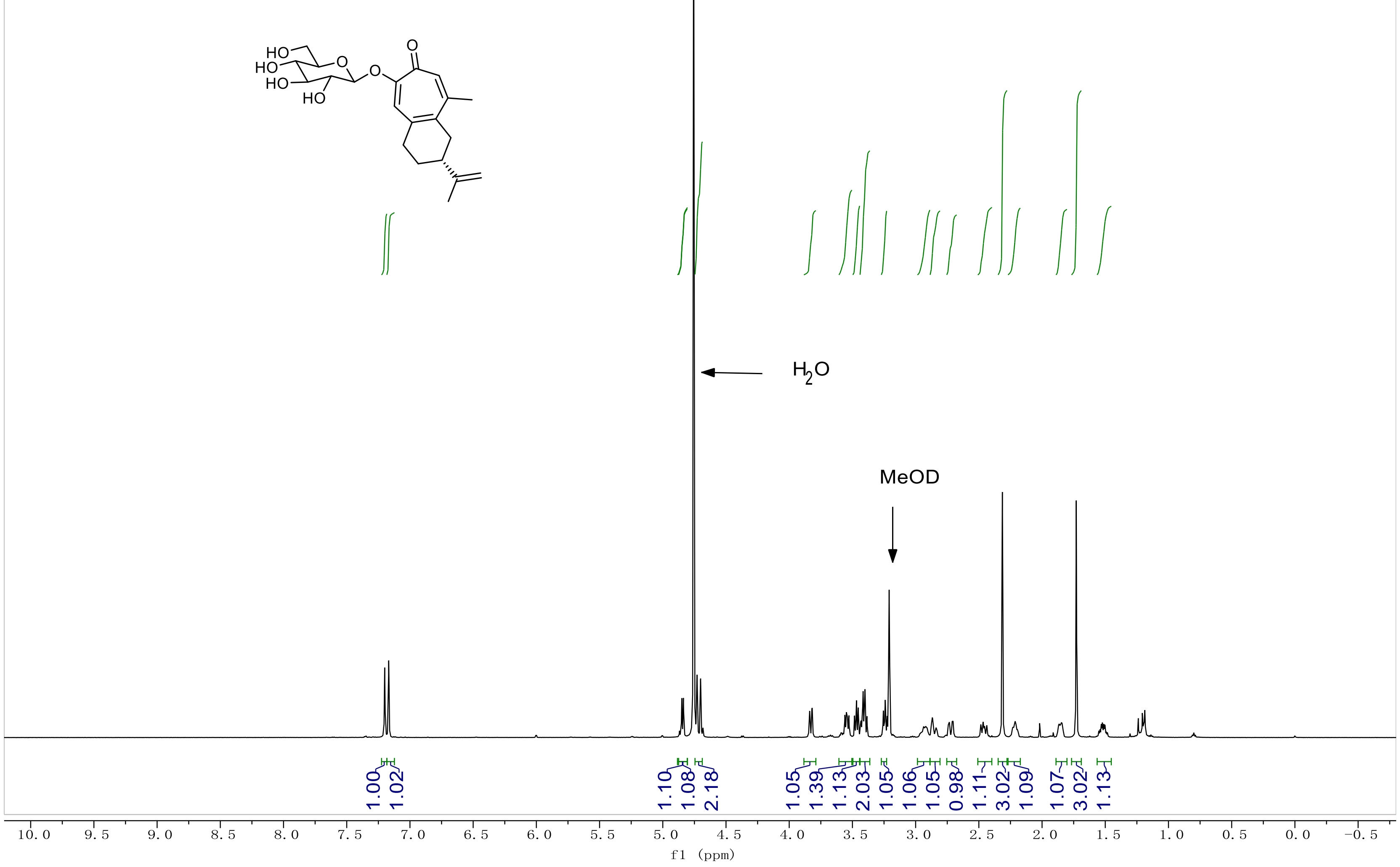

150 MHz $^{13}C\{^{1}H\}$ NMR Spectrum of Compound **5**
(recorded in CD_3OD)

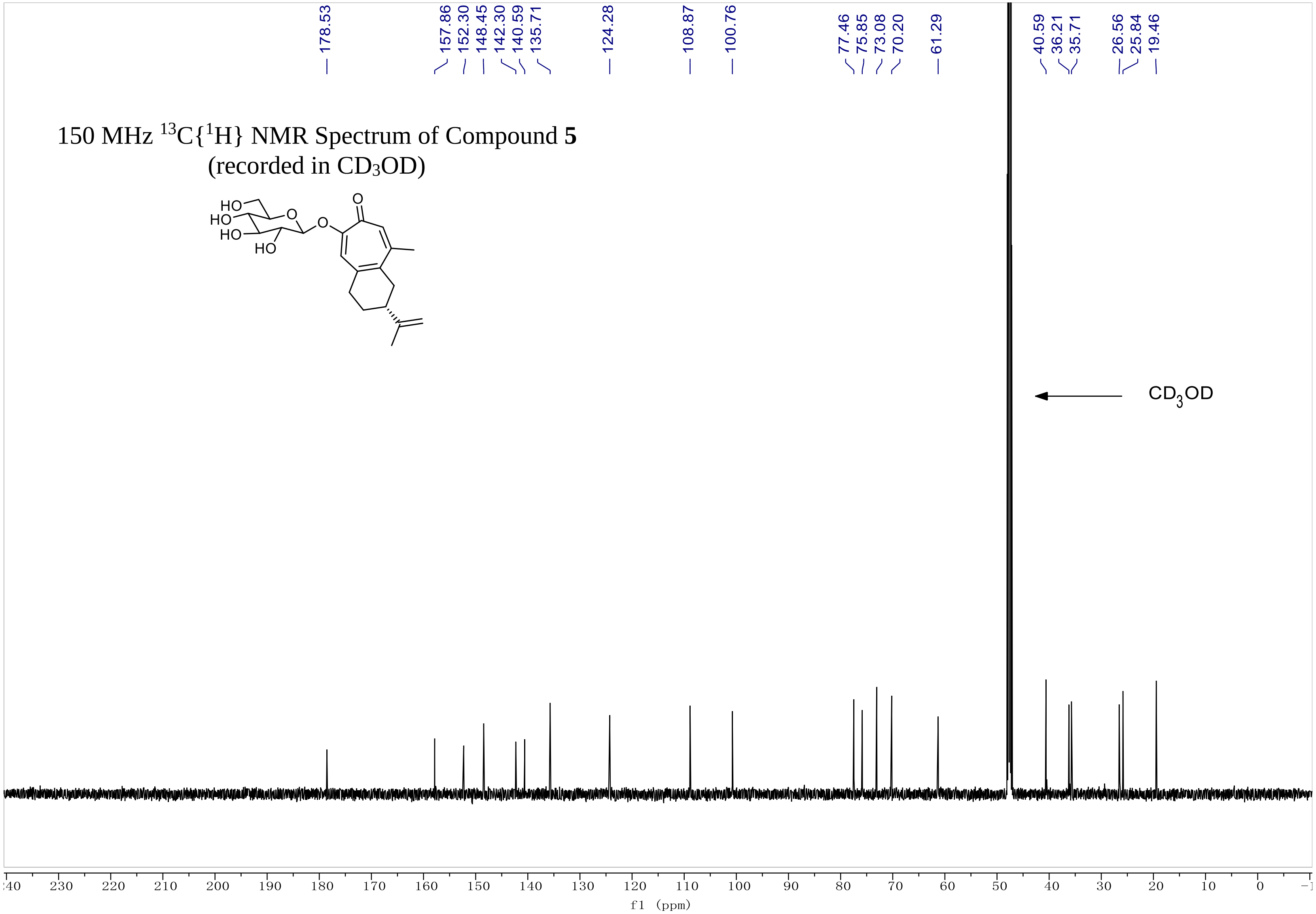

Supplement: Supplementary file 1 — Supporting Information [file OPEN-14-e202500011-s001.pdf]
